# An integrative analysis of single-cell and bulk transcriptome and bidirectional mendelian randomization analysis identified C1Q as a novel stimulated risk gene for Atherosclerosis

**DOI:** 10.3389/fimmu.2023.1289223

**Published:** 2023-12-21

**Authors:** Hong-Kai Cui, Chao-Jie Tang, Yu Gao, Zi-Ang Li, Jian Zhang, Yong-Dong Li

**Affiliations:** ^1^ Department of Neurological Intervention, The First Affiliated Hospital, Xinxiang Medical University, Xinxiang, Henan, China; ^2^ Institute of Diagnostic and Interventional Radiology, Shanghai Sixth People's Hospital Affiliated to Shanghai Jiao Tong University School of Medicine, Shanghai, China

**Keywords:** atherosclerotic plaque (AP), ScRNA-seq, Mendelian randomization (MR), complement component 1q (C1q), LASSO

## Abstract

**Background:**

The role of complement component 1q (C1Q) related genes on human atherosclerotic plaques (HAP) is less known. Our aim is to establish C1Q associated hub genes using single-cell RNA sequencing (scRNA-seq) and bulk RNA analysis to diagnose and predict HAP patients more effectively and investigate the association between C1Q and HAP (ischemic stroke) using bidirectional Mendelian randomization (MR) analysis.

**Methods:**

HAP scRNA-seq and bulk-RNA data were download from the Gene Expression Omnibus (GEO) database. The C1Q-related hub genes was screened using the GBM, LASSO and XGBoost algorithms. We built machine learning models to diagnose and distinguish between types of atherosclerosis using generalized linear models and receiver operating characteristics (ROC) analyses. Further, we scored the HALLMARK_COMPLEMENT signaling pathway using ssGSEA and confirmed hub gene expression through qRT-PCR in RAW264.7 macrophages and apoE-/- mice. Furthermore, the risk association between C1Q and HAP was assessed through bidirectional MR analysis, with C1Q as exposure and ischemic stroke (IS, large artery atherosclerosis) as outcomes. Inverse variance weighting (IVW) was used as the main method.

**Results:**

We utilized scRNA-seq dataset (GSE159677) to identify 24 cell clusters and 12 cell types, and revealed seven C1Q associated DEGs in both the scRNA-seq and GEO datasets. We then used GBM, LASSO and XGBoost to select C1QA and C1QC from the seven DEGs. Our findings indicated that both training and validation cohorts had satisfactory diagnostic accuracy for identifying patients with HPAs. Additionally, we confirmed SPI1 as a potential TF responsible for regulating the two hub genes in HAP. Our analysis further revealed that the HALLMARK_COMPLEMENT signaling pathway was correlated and activated with C1QA and C1QC. We confirmed high expression levels of C1QA, C1QC and SPI1 in ox-LDL-treated RAW264.7 macrophages and apoE-/- mice using qPCR. The results of MR indicated that there was a positive association between the genetic risk of C1Q and IS, as evidenced by an odds ratio (OR) of 1.118 (95%CI: 1.013–1.234, P = 0.027).

**Conclusion:**

The authors have effectively developed and validated a novel diagnostic signature comprising two genes for HAP, while MR analysis has provided evidence supporting a favorable association of C1Q on IS.

## Introduction

Atherosclerosis is a disease in which several cell types, such as SMCs, ECs, and immune cells, are activated pathophysiologically (1–3). Atherosclerosis progresses gradually over time, and as the lumen narrows, clinical symptoms such as angina and dizziness emerge. Eventually, this narrowing leads to an ischemic stroke or myocardial infarction due to plaque rupture or erosion (4, 5). Therefore, developing an accurate method for diagnosing HAP is crucial for early intervention.

Currently, the diagnosis of HAP heavily relies on clinical manifestations, functional outcomes, and invasive intravascular imaging techniques such as computed tomography angiography and magnetic resonance angiography that thoroughly assess vessel stenosis and wall thickness. Non-invasive medical imaging can accurately identify vulnerable plaques and stratify cardiovascular risk with the advancement of molecular biology and non-invasive molecular tools developed for the diagnosis and risk stratification of atherosclerotic plaques (6–14).

Atherosclerosis is a persistent inflammatory ailment that is distinguished by the buildup of macrophages that are laden with lipids in the vascular wall (15, 16). Numerous studies have shown that immune cell infiltration within the vessel wall is strongly associated with atherosclerosis initiation and progression (11, 17, 18). The immune composition of atherosclerotic plaque was inferred from multiple sources such as bulk RNA-seq data, CyTOF analysis of a single plaque, and scRNA-seq analysis of atherosclerotic tissue (17, 19–22). Single-cell RNA sequencing and time-of-flight cytometry have been utilized to analyze immune cell composition in both murine and human atherosclerotic plaques (17, 20). Bioinformatics analysis of immune cell infiltration using bulk RNA-seq data from atherosclerotic tissue has been previously explained (7–13). However, very few studies have been conducted utilizing scRNA-seq to predict HPA diagnosis (14, 23).

Recently, C1Q has been identified as a distinct subset of tissue resident macrophages, tumor-associated macrophages (TAMs) and tumor immune microenvironments (TIMs), and is commonly acknowledged as a facilitator of cancer progression (24–27). In the present study, we also identified a significant involvement of the C1Q cell cluster in the pathogenesis of atherosclerosis. Moreover, to gain deeper insights into the potential risk factor of C1Q in the development of HAP, we employed a statistical technique known as mendelian randomization (MR). MR utilizes single nucleotide polymorphisms (SNPs) to simulate randomized controlled trials, aiming to ascertain and explore the causal association between exposure and outcome variables in epidemiological research. By effectively eliminating the confounding effects of extraneous factors and employing genetic variations as instrumental variables (IVs), MR enables the analysis of disease relationships (28–32).

Therefore, our goal is to develop gene signatures utilizing the molecular characteristics of C1Q associated genes for the effective diagnosis and prediction of patients with human atherosclerotic plaques (HAP) and investigating the association between C1Q and ischemic stroke (large artery atherosclerosis) with MR. Initially, we analyzed scRNA-seq and bulk RNA-seq data of human atherosclerotic plaque (HAP) to identify immunomarkers for the diagnosis and prediction of HAP. Subsequently, we validated the diagnostic value of hub markers using GEO datasets and analyzed the relationship between the signatures and the landscape of immune cell infiltration. Additionally, we screened the potential transcription factors (TFs) that may regulate the hub genes in GEO datasets and examined both the hub genes and TFs in apoE-/- mice. Finally, we investigated the association between C1Q and IS (large artery atherosclerosis) using bidirectional MR analysis.

## Materials and methods

### Data availability

Single-cell transcriptome profiles of human carotid atherosclerotic plaques (HAP) and adjacent normal tissue samples were obtained from the Gene Expression Omnibus (GEO) database (accession code GSE159677). To supplement the analysis, we accessed bulk RNA-sequencing data from four other GEO datasets for atherosclerotic patients, namely GSE28829 (33), GSE43292 (34), GSE41571 (35), and GSE100927 (36), each containing over 10 patient cases (**Table S1**).

The GSE28829 dataset comprises 13 early and 16 advanced specimens of carotid atherosclerotic plaque that were detected using the Affymetrix Human Genome U133 Plus 2.0 Array. The GSE43292 dataset contains 32 early-stage and 32 advanced-stage specimens of carotid atherosclerotic plaque that were detected using the Affymetrix Human Gene 1.0 ST Array. The GSE41571 dataset has 5 ruptured and 6 stable specimens of atherosclerotic plaque that were detected using the Affymetrix Human Genome U133 Plus 2.0 Array. The GSE100927 dataset includes 35 healthy arteries, consisting of 12 carotid, 12 femoral, and 11 infra-popliteal territories arteries. Additionally, the dataset has 69 atherosclerotic arteries, which comprise 29 carotid, 26 femoral, and 14 infra-popliteal territories atherosclerotic arteries. All these were detected using the Agilent-039494 SurePrint G3 Human GE v2 8x60K Microarray. We divided the GSE100927 dataset, which initially contained three sets, into three subparts. These are GSE100927_Carotid that has 29 carotid atherosclerotic arteries and 12 carotid normal arteries; GSE100927_Femoral containing 26 femoral atherosclerotic arteries and 12 femoral normal arteries, and GSE100927_Infra that has 14 infra-popliteal territories atherosclerotic arteries and 11 infra-popliteal territories normal arteries.

The present research utilized a publicly available dataset with pre-existing ethics approval. Every participant gave their informed consent. The present study was conducted as per the principles of the Declaration of Helsinki.

### Identification of differential immune cell genes by scRNA-seq analysis

To analyze the ScRNA-seq data, we relied on the "Seurat" (version 4.1.2) and "SingleR" (version 1.6.1) packages (37). Initially, we eliminated low-quality cells by applying specific criteria, including minimum expression cells greater than 3, gene numbers less than 200, and mitochondrial genes exceeding 15%. The remaining cells were subjected to further bioinformatic analysis. To perform a principal component analysis (PCA), we selected the top 2,000 genes with the highest variability in expression. Subsequently, we utilized the T-distributed stochastic neighbor embedding (t-SNE) technique for dimension reduction based on the top 15 principal components. Ultimately, we identified significant marker genes with an adjusted p-value less than 0.05 and a |log2 (fold change)| greater than 1.

### Cell clustering analysis, visualization, and annotation

We performed cell clustering and sub-clustering analyses using the FindClusters function of the Seurat package, utilizing appropriate resolutions. We filtered cells with ribosome gene ratios higher than 15% for the re-clustering of each type of cell cluster. We utilized the Uniform Manifold Approximation and Projection (UMAP) technique to display the identified cell clusters and sub-clusters. We annotated the cell clusters with highly-expressed genes, marker genes from differential expression gene (DEG) analysis, and reported cellular markers. We applied the DEG analysis to all of the cell clusters using the FindAllMarkers function embedded in Seurat (version 4.1.2) to identify useful information marking the plaque state. We selected the top five genes based on their log2 fold-change value to serve as the initial feature input for machine learning.

### Functional enrichment analysis for scRNA-seq

Conducted a Gene Ontology (GO) and Kyoto Encyclopedia of Genes and Genomes (KEGG) analysis of the differential marker genes between subclusters using ClusterProfiler 4.0 in R (38). We used GSVA package (39) to analyze the differential marker genes among subclusters. All gene sets were obtained from the Molecular Signatures Database MSigDB (https://www.gseamsigdb.org/gsea/downloads.jsp) (40). We used the Scillus package to perform Gene Set Enrichment Analysis (GSEA) on the differential marker gene expression among subclusters (https://github.com/xmc811/Scillus).

### Immune landscape and signal pathways analysis

In this study, the immune microenvironment (IME) was assessed using various algorithms, namely EPIC, XCell, MCPCOUNTER, QUANTISEQ, IPS, ESTIMATE, and TIMER, which were obtained from the IMvigor210CoreBiologies R package (41). Additionally, the ssGSEA algorithm from the "GSVA" package was added to calculate infiltration scores for 29 immune cells (42). Furthermore, immunomodulators (43), including Antigen presentation, Cell adhesion, Co-inhibitor Co-stimulator, Ligand, Receptor, and Other, were estimated based on high and low C1Q expression level. Lastly, the enrichment of 16 signal pathways (44) in the three datasets was analyzed by the ssGSEA algorithm from the "GSVA" package using C1Q.

### Selection of characteristic immune gene and model construction

We employed Gradient Boosting Machine (GBM), logistic least absolute shrinkage and selection operator (LASSO) regression, and extreme gradient boosting (XGBoost) algorithms to identify C1Q-related hub genes (45). The diagnostic performance of our model was evaluated using receiver operating characteristic (ROC) curves and the area under the curve (AUC), employing a generalized linear model.

### *In vitro* cell analyses

RAW264.7 cells were cultured using DMEM supplemented with 10% FBS and 1% penicillin/streptomycin in an automatic incubator set to 37°C and 5% CO2. The cells were passaged or plated at 80-90% confluency. Then, the cells were seeded into 6-well plates at a density of 5000 cells per well. Following cell adhesion, 50ug/ml ox-LDL were added and incubated for 1, 3, 5 days, respectively. To extract RNA for qRT-PCR, total RNA was isolated from RAW264.7 macrophages using Trizol reagent and reverse transcribed with the Color Reverse Transcription Kit (EZB, USA).

### Atherosclerotic mouse model construction

Six-week-old male apoE-/- mice (Gempharmacy Co., Ltd, China) and six-week-old male C57/B6J wild-type mice (Gempharmacy Co., Ltd, China, no apoE-/- background) were housed individually in the experimental animal center's specific pathogen-free barrier system (SPF) of Shanghai Sixth People's Hospital Affiliated to Shanghai Jiao Tong University School of Medicine. The six apoE-/- knockout mice were fed a high-fat diet (68.3% chow diet, 31.7% lard) for 18 weeks, while the remaining six C57/B6J wild-type mice were used as the control group and were fed a chow diet for 18 weeks. At the end of the 18th week, the mice were euthanized, and blood samples were obtained from the abdominal aorta to measure lipid metabolism indexes. Both the thoracic and abdominal arteries of each mouse were dissected and harvested for real-time quantitative polymerase chain reaction (qPCR) analysis. Additionally, the aortic arch samples were collected, fixed in 4% paraformaldehyde (PFA) for at least 24 hours, and processed for immunohistochemistry analysis.

### Quantitative real-time PCR

Total cellular RNA was extracted following the instructions of the manufacturer and using a TRIzol reagent (Invitrogen, USA). Subsequently, cDNA was generated through reverse transcription of RNA, employing PrimeScript RT Master Mix (Takara). For the thoracic and abdominal artery tissues, the primer sequences used were (5’-3'): GAPDH (forward: 5'-CCTCGTCCCGTAGACAAAATG-3', reverse: 5’- TGAGGTCAATGAAGGGGTCGT-3), C1QA (forward: 5'- TCACCAACCAGGAG- AGTCCA -3', reverse: 5'- CACCTGAAAGAGCCCCTTGT- 3'), C1QC (forward: 5'- GCCGATACAAA- CAGAAGCACCA-3', reverse: 5'- AACTTCCCTGTGCTTGG- GTTGT-3'), SPI1 (forward: 5'- TTTGAGAACTTCCCTGAGAACCAC-3', reverse: 5’- GCATG TAGGAAACCTGGTGACT- G-3'), IL1B (forward: 5'- GCATCCAG- CTTCAAATCTCGC-3', reverse: 5'- TGTTCATCTCG- GAGCCTGTAGTG- 3'), ABCG1 (forward: 5'- TTGTGCTGTTCGCTGCTCTG-3', reverse: 5'- GTCACGG- GACCCACAAATGT-3') synthesized by shanghai Generay Biotech. The primer sequence was obtained from Primer Bank (https://pga.mgh.harvard.edu/primerbank/index.html) and synthesized by Shanghai Sangon Biotechnology (Shanghai, China). The GAPDH gene was used as an internal reference gene. Calculation of the expression of the target gene was performed based on the 2-ΔΔCt method.

### Statistical analysis

We conducted statistical analysis using R software (version 4.2.1). Mean and standard deviation (SD) were used to present the data. We used Wilcoxon or Student's t-test to compare the difference between two groups. For comparing three or more groups, we employed Student t-test and analyzed variance (ANOVA). To determine the correlation between variables, we used either Pearson's or Spearman's correlation tests. The statistical p-values we used were two-sided, and we considered p-values where p < 0.05 as statistically significant.

### Mendelian randomization analysis

#### Study design and data source

In order to assess the correlation between C1Q and ischemic stroke, specifically in relation to large artery atherosclerosis, we conducted datasets of relevant diseases from the IEU openGWAS (https://gwas.mrcieu.ac.uk). Given that the data originated from publicly accessible databases, no supplementary ethical clearance was deemed necessary. The study utilized GWAS data encompassing 4373 cases of ischemic stroke (large artery atherosclerosis) and 406,111 control cases, and C1Q protein were also obtained from GWAS data for the study (**Table S8**). The study utilized samples exclusively derived from individuals of European descent. A bidirectional two-sample Mendelian randomization (MR) investigation was conducted to examine the potential relationship between C1Q and IS. MR was employed to assess the potential association between C1Q and IS using carefully selected instrumental variables (IVs). Additionally, sensitivity analysis was performed to evaluate the robustness of the findings. Lastly, reverse causality verification was undertaken to obtain a comprehensive analysis report. **Figure S6** provides a visual representation of the Mendelian randomization study investigating the relationship between C1Q and IS.

#### Statistical analysis for Mendelian randomization

The data analysis for this study was conducted utilizing the R software (version 4.2.2) with the TwoSampleMR package (0.5.7), following the guidelines provided at https://mrcieu.github.io/TwoSampleMR/. Mendelian randomization (MR) employs genetic variation as an instrumental variable (IV) to estimate the causal effect of risk factors on complex diseases. We conducted a genome-wide association study (GWAS) to identify single nucleotide polymorphisms (SNPs) that were both independent and nominally associated with a significance threshold of P < 1×10-5. We used a clumping algorithm with a cutoff of r2 = 0.01 and kb = 10000 to select SNPs that were in linkage disequilibrium with each other (28, 29). Previous studies have described the methods used for other Mendelian randomization (MR) analyses (30–32). To assess heterogeneity among instrumental variables (IVs), we employed Cochran's Q test, with a P-value greater than 0.05 indicating no significant heterogeneity. We considered an IV to exhibit horizontal pleiotropy if the MR-Egger regression intercept was not equal to 0 and had a statistically significant P-value less than 0.05. In the reverse MR analysis, a P-value greater than 0.05 indicated no evidence of reverse causality between the exposure factors and the outcome variables.

## Results

### Profiling of scRNA-Seq and screening of marker genes

The workflow of the study design is depicted in **Figure S1**. ScRNA-seq data were obtained from plaque specimens and adjacent tissues of the patient who underwent carotid endarterectomy (CEA). Three patients provided plaque specimens from near full-thickness sections of arteries and plaques from the atherosclerotic core (AC: patient 1-3), as well as adjacent tissue from full-thickness proximally adjacent (PA: patient 1-3) arterial sections. The proximally adjacent (PA) tissues were deemed to be the normal tissue. A total of 50,856 cell samples from six patients with HAP were retrieved from the GEO database containing 38,611 AC cells and 12,245 PA cells (**Table S2**). The number of cells in AC was three times greater than that in PA. Following initial quality control, 44,843 cells including 34,378 cells and 10,465 normal cells from all six samples were used for further analysis of their single-cell transcriptomic data (**Table S3**). The number of cells from the AC region was significantly higher than those analyzed by Li et al. (46), as we analyzed the AC and PA data together.

**Figure 1:**
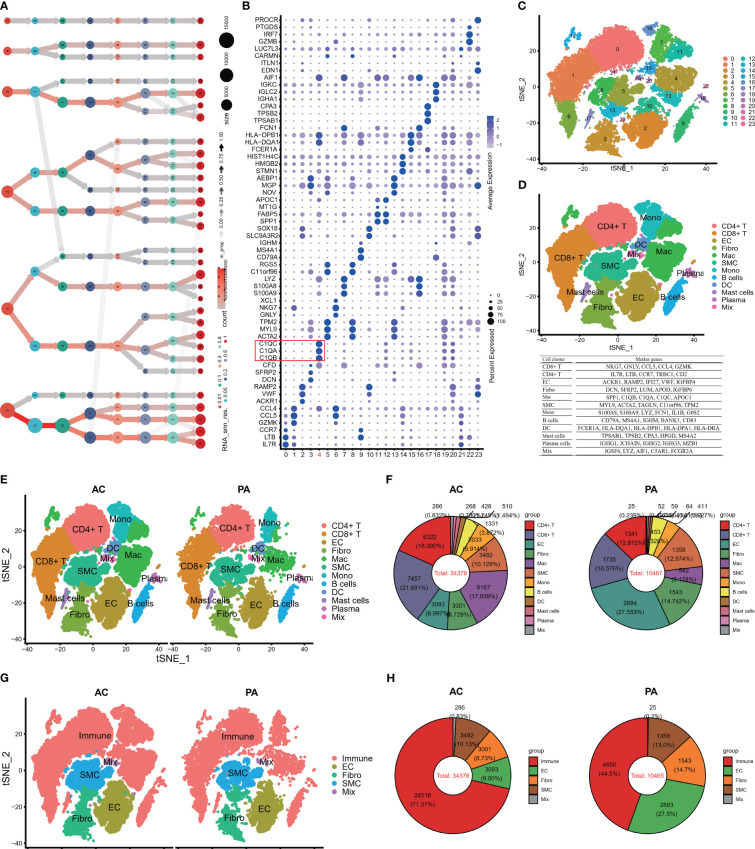
Single-cell RNA-seq of human AP tissues. **(A)** The clustering tree of total scRNA-seq mate data was analyzed at different resolutions. **(B)** The top three markers of each cluster were plotted using the “FindAllMarkers” function from the Seurat package (4.1.2). The red box indicates C1Q cell cluster. **(C)** T-distributed stochastic neighbor embedding (tSNE) revealed 24 clusters under a resolution of 0.8. **(D)** The tSNE plot was colored to display 12 distinct cell types. Note: The marker gene is located below the tSNE plot. **(E)** An overview of the 12 cell types between AC and PA groups was generated and colored by cell types. **(F)** Using a pie plot, the proportion of cell types in each group was compared. **(G, H)** The tSNE and pie plots were used to depict cell types between AC and AP groups after merging the immune cells with the Seurat package (4.1.2). AC, Atherosclerotic core; PA, Proximally adjacent; EC, Endothelial cells; Mac, Macrophages; SMC, Smooth muscle cells; Mono, Monocytes; DC, Dendritic cells.

Following batch effect correction and normalization through the Harmony R package, the evaluation yielded twenty-four major cell clusters that exhibited distinct gene expression patterns at a resolution of 0.8 (**Figures 1A–C**). The genes in twenty-four cell clusters were shown in **Table S4**. The clusters were then combined to form a total of 12 cell clusters, which comprised of 8 immune cell subtypes (CD8+ T, CD4+ T, macrophage, monocyte, B cells, mast cells, plasma cells, and dendritic cells), 3 non-immune cell subtypes (SMC, EC, and Firboblast clusters), and a miscellaneous cell cluster (**Figure 1D**). The FindAllMarkers embedded in Seurat were then applied to all the cell clusters to search for DEGs to compare all of the cell clusters (**Table S5**; **Figure 1D**). Finally, we performed FindMarkers to estimate DEGs between AC and PA, and 781 DEGs were obtained (**Table S6**).

Several studies have demonstrated the involvement of immune cell infiltrations and pathways in atherosclerosis, highlighting their crucial role in atherosclerotic plaque progression (9, 11, 17, 47, 48). **Figures 1E, F** illustrates that in the PA group, endothelial cells (EC) and clusters of CD8+ T cells and fibroblasts constituted the top three cell clusters, whereas in the AC group, CD8+ T, CD4+ T, and macrophages were predominant. When considering all immune cell types together, namely CT4+ T, CT8+ T, macrophages, monocytes, dendritic cells, B cells, mast cells, and plasma cells, they accounted for more than 70% of the total cells in the AC groups (**Figures 1G, H**), whereas immune cells made up less than 45% of the total cells in the PA groups (**Figures 1G, H**). These findings indicate that immune cell involvement in atherosclerotic plaque progression increased significantly.

### C1Q hub genes selection from GEO and scRNA-seq

**Figure 2:**
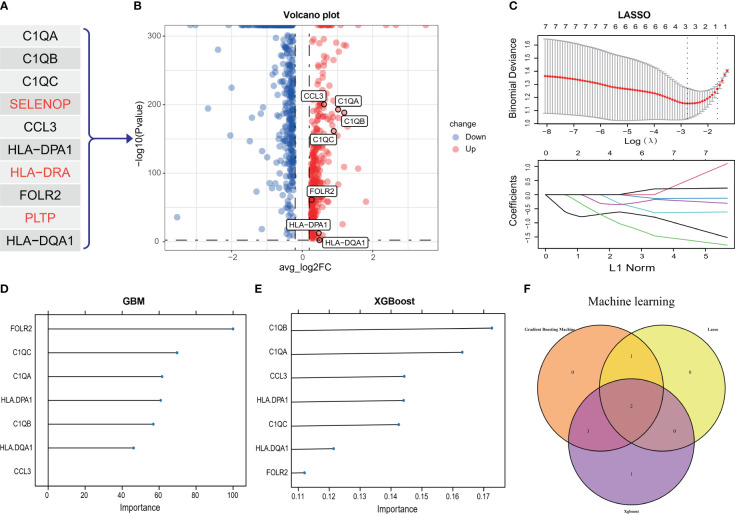
C1Q hub genes selection from scRNA-seq and GEO dataset. **(A)** The top ten genes extract from C1Q cell cluster. **(B)** The 10 genes are detected in 781 DEGs between AC and PA groups in scRNA-seq, and seven genes (C1QA, C1QB, C1QC, CCL3, HLA-DPA1, FOLR2 and HLA-DQA1) was obtained for further analysis. **(C)** C1Q hub genes selection by LASSO algorithm. **(D)** C1Q hub genes selection by GBM algorithm. **(E)** C1Q hub genes selection by XGBoost algorithm. **(F)** Two genes (C1QA and C1QC) are identified by the three algorithms.

Two steps were applied to select C1Q-associated hub genes. First, the top 10 genes in C1Q subcluster were extracted as initial features (**Figure 2A**). And then, we detected the 10 genes in 781 DEGs between AC and PA groups in scRNA-seq (**Figure 2B**), and seven DEGs (C1QA, C1QB, C1QC, CCL3, HLA-DPA1, FOLR2 and HLA-DQA1) was obtained for further analysis. Second, the dataset GSE43292 was utilized to select characteristic genes in atheroma plaque (stage IV and over) and distant macroscopically intact tissue (stages I and II) using machine learning models (LASSO (**Figure 2C**) Gradient Boosting Machine (**Figure 2D**), and XGBoost (**Figure 2E**)). The three algorithm was used to reduce the number of biomarkers, resulting in the selection of 2 biomarkers (C1QA and C1QC), as shown in **Figure 2F**. Hence, the two biomarkers were identified as the definitive diagnostic prediction biomarkers.

### The expression and signaling pathways involved in characteristic genes in scRNA-seq

**Figure 3:**
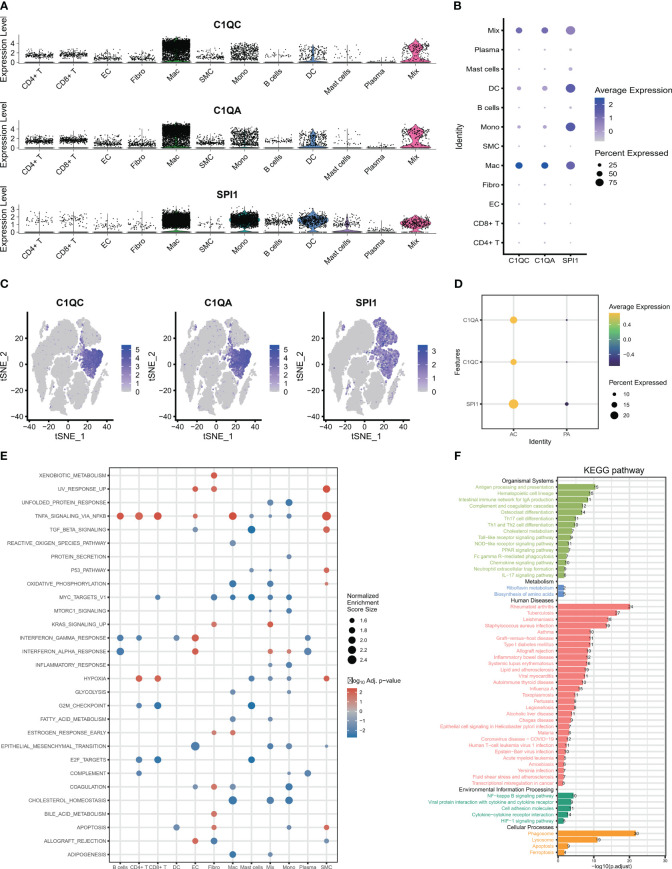
The expression and signaling pathways involved in characteristic genes in scRNA-seq. **(A–C)** The plots display the expression of C1QA, C1QC and SPI1 in cell clusters using scRNA-seq. **(D)** The three characteristic genes were upregulated in AC group. **(E)** The GSEA showed that the signaling pathways in all 12 cells clusters. **(F)** The KEGG plot shows the KEGG pathway in macrophage clusters.

After identifying the biomarker genes, we prioritized investigating their expression in HAP cells. As displayed in **Figures 3A–C**, C1QA and C1QC were mainly expressed in macrophage cells, and upregulated in AC group (**Figure 3D**), while SPI1 was expressed in macrophages, monocytes, dendritic cells (**Figures 3A–C**), and also upregulated in AC group (**Figure 3D**). Subsequently, we conducted GSEA for all the identified cells. The GSEA demonstrated significant enrichment of crucial signaling pathways in HAP tissues (**Figure 3E**). These signaling pathways include immune signaling (INTERFERON_GAMMA_ RESPONSE, IL6_JAK_STAT3_SIGNALING, IL2_STAT5_SIGNALING, COMPLEMENT), allograft rejection, complement, inflammatory response, metabolism, apoptosis, and glycolysis. We conducted KEGG pathway enrichment analysis for macrophages derived from all differentially expressed genes between AC and PA. KEGG pathway analysis revealed the enrichment of various immune pathways involving Th17 cell differentiation, as well as the differentiation of Th1 and Th2 cells, toll-like receptor signaling pathway, and complement and coagulation cascades, among others (**Figure 3F**). These results suggest that multiple immune pathways and signals contribute to the pathophysiological progression of atherosclerotic plaque.

### Efficient diagnostic model development and validation for HAP tissues and adjacent tissues

**Figure 4:**
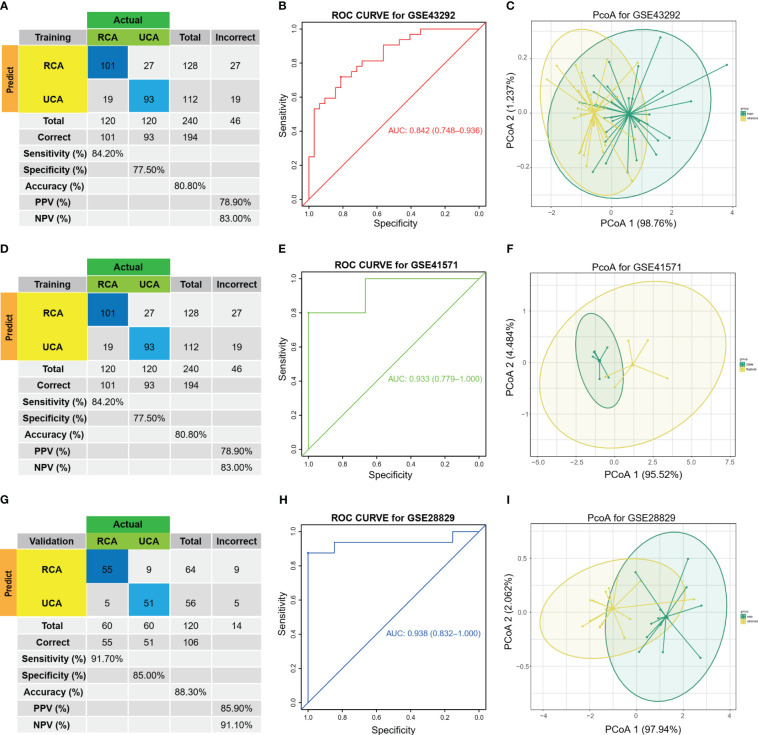
Diagnostic prediction model for AP progression. **(A)** The actual and predicted samples with confusion matrices built from the diagnostic prediction model using the two biomarkers with generalized linear models (regressions) between Atheroma and intact in the GSE43292 training cohorts. **(B)** Assessment of the diagnostic predictive accuracy for the training cohort for the two signatures using ROC curves (Atheroma = 32, intact = 32, AUC = 0.842). **(C)** PCoA analysis showing that the two signatures can distinguish between Atheroma and intact samples significantly. **(D)** The actual and predicted samples with confusion matrices built from the diagnostic prediction model using the two biomarkers with generalized linear models (regressions) in the GSE41571 external validation cohorts. **(E)** Assessment of the diagnostic predictive accuracy for the validation cohort for the two signatures using ROC curves (Ruptured = 5, Stable = 6, AUC = 0.933). **(F)** PCoA analysis showing that the two signatures can discriminate ruptured from stable samples significantly. **(G)** The actual and predicted samples with confusion matrices built from the diagnostic prediction model using the two biomarkers in the GSE28829 external validation cohorts. **(H)** Assessment of the diagnostic predictive accuracy for the validation cohort for the two signatures using ROC curves (advanced = 13, early = 16, AUC = 0.938). **(I)** PCoA analysis showing that the two signatures can discriminate advanced from early samples significantly.

After selection of the immune DEGs, we trained and validated our model using GEO data. GSE43292 was used as training datasets, respectively, while GSE41571 and GSE28829 was used as an external validation dataset to develop and validate the signatures for diagnosing and predicting atherosclerotic plaque progression between HAP tissues and adjacent tissues. Firstly, a model was built with two biomarkers using a generalized linear model (regression). The model score was -24.304 + (1.169 * C1QA + 1.339 * C1QC) (**Table S7**). Fitting the two markers into the model revealed high consistency between predicted results and surgical diagnosis results in the training dataset (**Figure 4A**). The AUC of the ROC curve was 0.842 while the specificity and sensitivity were 71.9% and 81.2%, respectively. This indicates good diagnostic efficiency in predicting atherosclerosis progression (**Figure 4B**). PCoA visualization was used with signatures and revealed a significant difference between HAP and adjacent tissues (**Figure 4C**).

The two biomarkers and the same statistical model were then used to evaluate the accuracy of the signature in predicting atherosclerotic plaque progression in the external validation cohorts (GSE41571 & GSE28829). Furthermore, the model demonstrated high consistency between the predicted and surgical diagnosis results in both validation cohorts, as depicted in **Figures 4D, G**. Additionally, the two biomarkers yielded satisfactory diagnostic accuracy in identifying patients with HPAs, with respective AUCs of 0.933 (95% CI: 0.779–1.0) for GSE41571 and 0.938 (95% CI: 0.832–1.0) for GSE28829. These results are depicted in **Figures 4E, H**. Similarly, there was a significant difference between the two groups on both validation cohorts, as shown in **Figures 4F, I**, when using PCoA.

The expression levels of the two immune biomarkers in HAP tissues and adjacent tissues were compared in the three GEO datasets. The results, shown in **Figures S2A–C**, demonstrated significant differences between the two groups across all three datasets. Furthermore, **Figures S2D–F** demonstrates strong correlations between the two immune biomarkers in all three datasets.

### External validation for diagnosis and predicting HAP from normal controls

**Figure 5:**
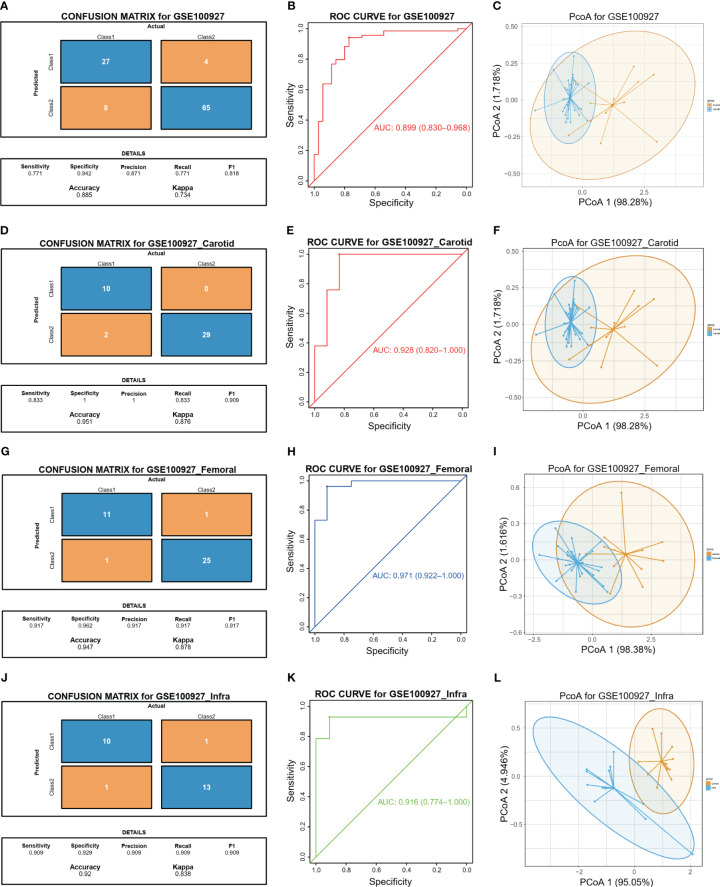
Diagnostic prediction model for diagnosis and predicting HAP from normal controls. **(A)** The actual and predicted samples with confusion matrices built from the diagnostic prediction model using the two biomarkers in the GSE100927 external validatin cohorts. **(B)** Assessment of the diagnostic predictive accuracy for the validation cohort for the two signatures using ROC curves (atherosclerotic arteries = 69, normal arteries = 35, AUC = 0.899). **(C)** PCoA analysis showing that the two signatures can distinguish between atherosclerotic arteries and normal arteries significantly. **(D)** The actual and predicted samples with confusion matrices built from the diagnostic prediction model using the two biomarkers in the GSE100927_Carotid external validatin cohorts. **(E)** Assessment of the diagnostic predictive accuracy for the validation cohort for the two signatures using ROC curves (Carotid = 29, Normal = 12, AUC = 0.928). **(F)** PCoA analysis showing that the two signatures can distinguish between carotid atherosclerotic arteries and normal arteries significantly. **(G)** The actual and predicted samples with confusion matrices built from the diagnostic prediction model using the two biomarkers in the GEO100927_Femoral external validation cohorts. **(H)** Assessment of the diagnostic predictive accuracy for the validation cohort for the two multi-omics signatures using ROC curves (Femoral = 26, Normal = 12, AUC = 0.981). **(I)** PCoA analysis showing that the two signatures can discriminate AP in femoral artery from normal samples significantly. **(J)** The actual and predicted samples with confusion matrices built from the diagnostic prediction model using the two biomarkers in the GSE100927_Infra validation cohorts. **(K)** Assessment of the diagnostic predictive accuracy for the validation cohort for the two signatures using ROC curves (Infra-popliteal territories = 14, Normal = 11, AUC = 0.89). **(L)** PCoA analysis showing that the two signatures can discriminate AP in infra-popliteal artery from normal samples significantly.

The study proceeded to perform an external validation of the efficient machine learning models for GSE100927, which included 69 atherosclerotic arteries and 35 healthy normal arteries from three different artery types, namely GSE100927_Carotid, GSE100927_Femoral, and GSE100927_Infra. To assess the validity of the method and the precision of the results, the study employed the two biomarkers and the same statistical model for GSE100927 and its three subsets. The model continued to exhibit a high level of consistency between the predicted outcomes and the surgical diagnosis for all four validation cohorts (**Figures 5A, D, G, J**). The two biomarkers demonstrated satisfactory diagnostic accuracy in identifying HPAs patients from normal patients, with the Area Under the Curve (AUCs) values of 0.899 for GSE100927 (**Figure 5B**), 0.928 for GSE100927_Carotid (**Figure 5E**), 0.971 for GSE100927_Femoral (**Figure 5H**), and 0.916 for GSE100927_Infra (**Figure 5K**). **Figures 5C, F, I, L** presented that the two groups could consistently be distinguished by utilizing the two signatures across all four datasets.

Last, the expression of the two immune biomarkers between HAP tissues and normal tissues in the three GEO datasets were presented at **Figures S3A–D**, and the significant differences between the two groups were observed in all datasets. In addition, the correlations between the two immune biomarkers were displayed in **Figures S3E–H**, and strong correlations were found in all datasets.

Finally, the expression levels of the two immune biomarkers in HAP tissues and normal tissues were compared in the three GEO datasets. The results, shown in **Figures S3A–D**, demonstrated significant differences between the two groups across all three datasets. Furthermore, **Figures S3E–H** demonstrates strong correlations between the two immune biomarkers in all three datasets.

### Immune microenvironment analysis based on C1Q hub genes

**Figure 6:**
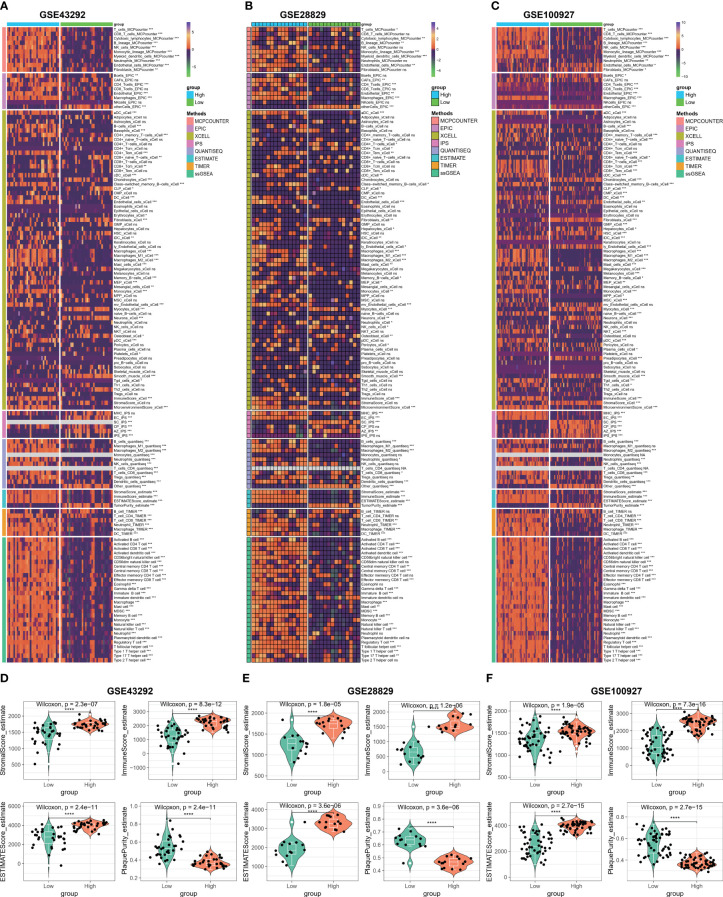
Immune microenvironment analysis based on C1Q hub genes. **(A)** Heatmap displaying enrichment of immune-infiltrating cells through 8 algorithms between atheroma and intact samples in the GSE43292 cohorts. **(B)** Heatmap displaying enrichment of immune-infiltrating cells through 8 algorithms between early and advanced samples in the GSE28829 cohorts. **(C)** Heatmap displaying enrichment of immune-infiltrating cells through 8 algorithms between atherosclerotic plaques and control samples in the GSE100927 cohorts. **(D–F)** The stromal scores, immune scores, ESTIMATE scores, and plaque purity are compared between high- and low C1Q groups in the GSE43292 **(D)**, GSE28829 **(E)**, and GSE100927 **(F)** datasets. *P <0.05, **P < 0.01, ***P < 0.001.

In order to further investigate the correlation between C1Q and the immune microenvironment of the HAP samples, a comparative analysis was conducted on the differences in immune cells between normal/early samples and HAP samples in three GEO datasets using eight algorithms (**Figures 6A–C**). The results revealed that a majority of the immune cells in the HAP samples exhibited distinct characteristics compared to the normal/early samples across the three GEO datasets. Furthermore, patients in the HAP group displayed significantly higher stromal scores, ESTIMATE scores, and immune scores, while the plaque purity was found to be lower than that of the normal or early group (P < 0.001) (**Figures 6D–F**). The findings of this study suggest that the HAP tissue obtained from the subgroup with high C1Q levels exhibited a greater abundance of immune cells and immune molecules.

### Immune signaling pathway and immunomodulators

**Figure 7:**
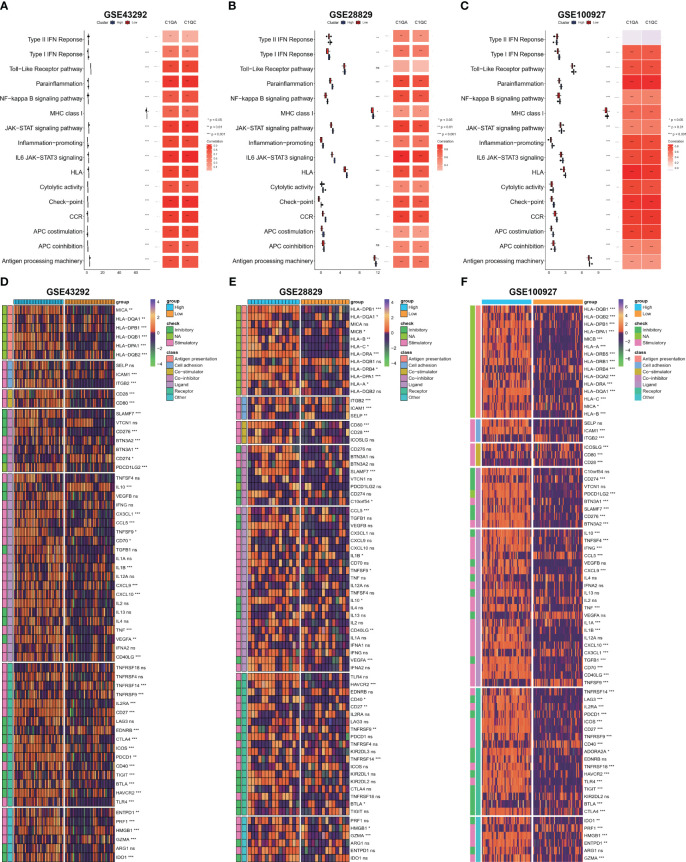
Evaluation of immune signaling pathway and immunomodulators based on C1Q hub genes. **(A–C)** Comparison of 16 immune signaling pathway between high- and low C1Q groups and correlation analysis between immune signaling pathway and C1QA or C1QC in the GSE43292 **(A)**, GSE28829 **(B)** and GSE100927 **(C)** datasets. **(D–F)** The enrichment of immunomodulators is visualized through heatmap analysis using seven algorithms in the same datasets: GSE43292 **(D)**, GSE28829 **(E)**, and GSE100927 **(F)**.

Based on our observations, it has been noted that HAP tissue with a high level of C1Q contains a greater number of immune cells and immune molecules. In order to further investigate this phenomenon, we proceeded to examine the immune signaling pathway and immunomodulators based on C1Q hub genes. The results, as depicted in **Figures 7A–C**, indicate that the majority of the 16 immune signal pathways were found to be enriched in the high C1Q group in HAPs. Furthermore, a positive correlation was observed between the majority of C1QA and C1QC and the 16 immune signal pathways in three GEO datasets. Additionally, the heatmap in **Figures 7D–F** illustrates that HAP samples with a high C1Q level exhibited significantly higher levels of immunomodulators levels. The findings of this study suggest that the HAP tissue obtained from individuals with high levels of C1Q exhibited a greater enrichment of immune signal pathways and a higher abundance of immunomodulators.

### C1QA and C1QC activated the HALLMARK_COMPLEMENT signaling pathway in HAP

**Figure 8:**
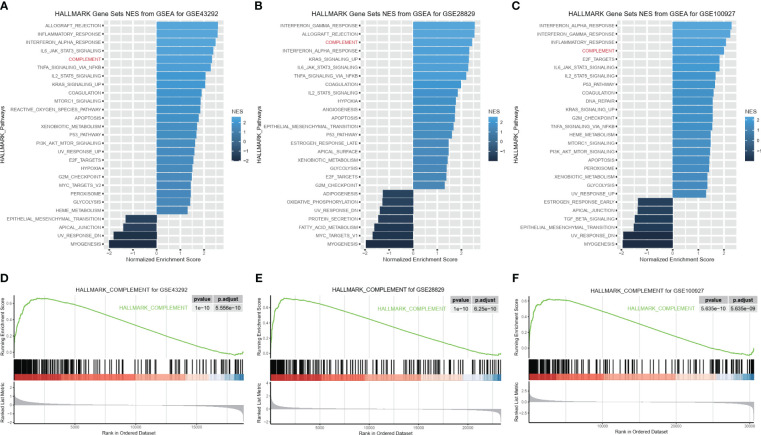
C1QA activated HALLMARK_COMPLEMENT signaling pathway in HAP. **(A–F)** GSEA analysis results of C1QA for three GEO datasets (GSE43292, GSE28829 and GSE100927).

To explore the molecular function of HAP in detail, we investigated whether C1QA and C1QC activate the HALLMARK_COMPLEMENT signaling pathway in GEO datasets. We conducted GSEA analysis on three GEO datasets (GSE43292, GSE28829, and GSE100927) to identify any signaling pathway that might be altered between AP and control samples. As anticipated, the HALLMARK_COMPLEMENT signaling pathway was activated in all three datasets (**Figures 8A–F**), indicating its crucial implication in HAP.

### Correlation between C1QA and C1QC and HALLMARK_COMPLEMENT signaling pathway in HAP

**Figure 9:**
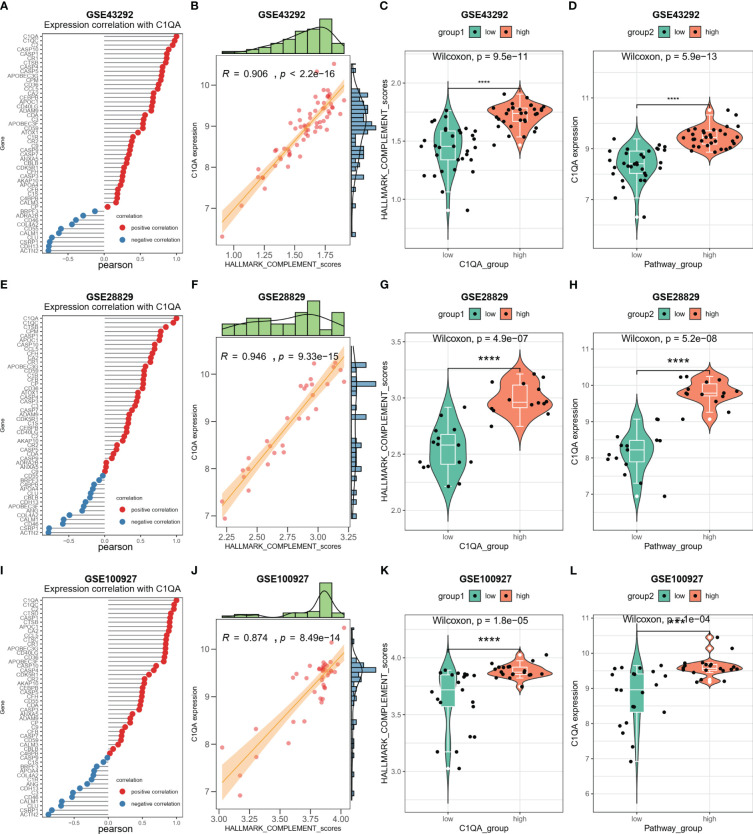
C1QA correlated HALLMARK_COMPLEMENT signaling pathway in HAP. **(A)** Correlation between C1QA and HALLMARK_COMPLEMENT signaling pathway genes in the GSE43292 dataset. **(B)** Correlation between C1QA and HALLMARK_COMPLEMENT signaling pathway scores in the GSE43292 dataset. **(C)** Comparison of the HALLMARK_COMPLEMENT signaling pathway scores between subgroups with high and low C1QA expression in the GSE43292 dataset. **(D)** Comparison of the expression of C1QA between subgroups with high and low HALLMARK_COMPLEMENT signaling pathway scores in the GSE43292 dataset. **(E)** Correlation between C1QA and HALLMARK_COMPLEMENT signaling pathway genes in GSE28829 dataset. **(F)** Correlation between C1QA and HALLMARK_COMPLEMENT signaling pathway scores in GSE28829 dataset. **(G)** Comparison of the HALLMARK_COMPLEMENT signaling pathway scores between high and low C1QA expression subgroups in GSE28829 dataset. **(H)** Comparison of C1QA expression between high and low subgroups of HALLMARK_COMPLEMENT signaling pathway scores in GSE28829 dataset. **(I)** Correlation between C1QA and HALLMARK_COMPLEMENT signaling pathway genes in GSE100927 dataset. **(J)** Correlation between C1QA and HALLMARK_COMPLEMENT signaling pathway scores in GSE100927 dataset. **(K)** Comparison of the HALLMARK_COMPLEMENT signaling pathway scores between high and low C1QA expression subgroups in GSE100927 dataset. **(L)** Comparison of C1QA expression between high and low subgroups of HALLMARK_COMPLEMENT signaling pathway scores in GSE100927 dataset.

The activation of the HALLMARK_COMPLEMENT signaling pathway by C1QA and C1QC in HAP was investigated. Subsequently, a correlation analysis was conducted between C1QA/C1QC and the HALLMARK_COMPLEMENT signaling pathway using three GEO datasets. Notably, our analysis revealed a positive correlation between C1QA and the majority of genes in this pathway (**Figures 9A, E, I**). Specifically, C2, CD36, CTSB, APOC1, CCL5, and others exhibited a high correlation with C1QA across all three datasets. Furthermore, the ssGSEA algorithm was employed to score the HALLMARK_COMPLEMENT signaling pathway for the three GEO datasets. Based on the obtained scores, a distinct positive correlation was observed between C1QA and the HALLMARK_COMPLEMENT signaling pathway (**Figures 9B, F, J**). The present study demonstrates the ability to discriminate pathway scores for subgroups with high and low C1QA expression (**Figures 9C, G, K**), as well as to distinguish C1QA expression levels between subgroups with high and low pathway scores (**Figures 9D, H, L**). These findings suggest that heightened C1QA expression is implicated in the progression of AP via the HALLMARK_COMPLEMENT signaling pathway. Furthermore, a comparable positive correlation between C1QC and the HALLMARK_COMPLEMENT signaling pathway was identified in HAP (**Figure S5**).

### Screening the key transcription factors regulating the two hub genes

**Figure 10:**
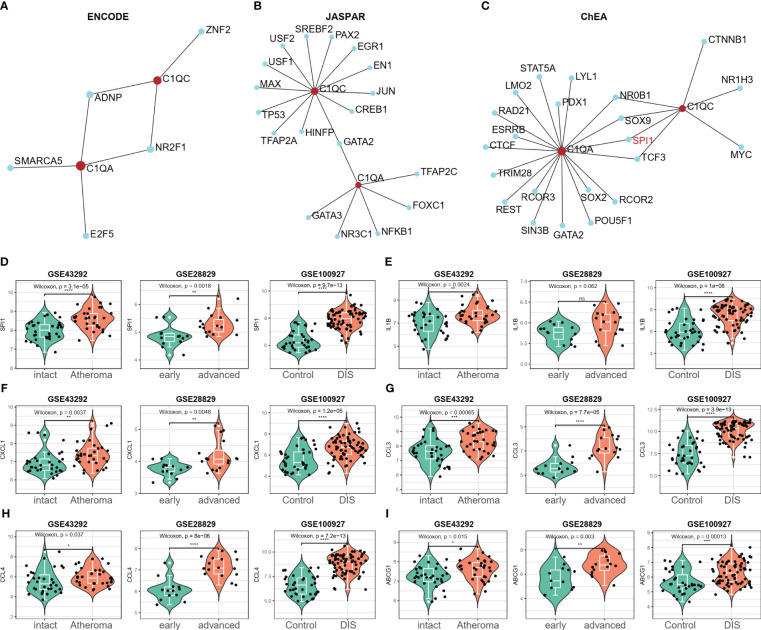
SPI1 was identified as a potential key TF in HAP. **(A–C)** The potential TFs that may regulate the C1QA and C1QC gene was screened from three databases (ENCODE, JASPAR and ChEA) via the NetworkAnalyst 3.0. **(D)** Only the expression of SPI1 significantly elevated in all the three GEO datasets (GSE43292, GSE28829 and GSE100927), and regarded as the potential TFs for C1QA and C1QC genes. **(E–I)** The expression of IL-1β, CXCL1, CCL3, CCL4 and ABCG1genes were upregulated in all the three GEO datasets.

The potential transcription factors (TFs) that could regulate the two crosstalk genes C1QA and C1QC were first screened from three databases (ENCODE, JASPAR, and ChEA) by using NetworkAnalyst 3.0. **Figures 10A–C** presents the TFs that could regulate the two crosstalk genes from the ENCODE, JASPAR, and ChEA databases as 2, 1, and 4, respectively. The mRNA expression levels of these TF were calculated in three GEO datasets, and as a result, the expression levels of SPI1 were considerably upregulated in all three datasets (**Figure 10D**). Hence, it can be concluded that SPI1 could be the crucial TF regulating the two crosstalk genes in HAP. Due to cytokines interleukin-1β (IL-1β) is considered to be the key mediators of HAP (49), and lipid metabolism related genes, such as ABCG1, etc., were the key to the transformation of macrophages into foam cells (50), hence we examined the expression levels of IL-1β, CXCL1, CCL3, CCL4, and ABCG1 in the three GEO datasets. **Figures 10E–I** shows that the expression levels of IL-1β, CXCL1, CCL3, CCL4, and ABCG1 were upregulated in all three GEO datasets.

### *In vitro* and *in vivo* analyses C1QA and C1QC

**Figure 11:**
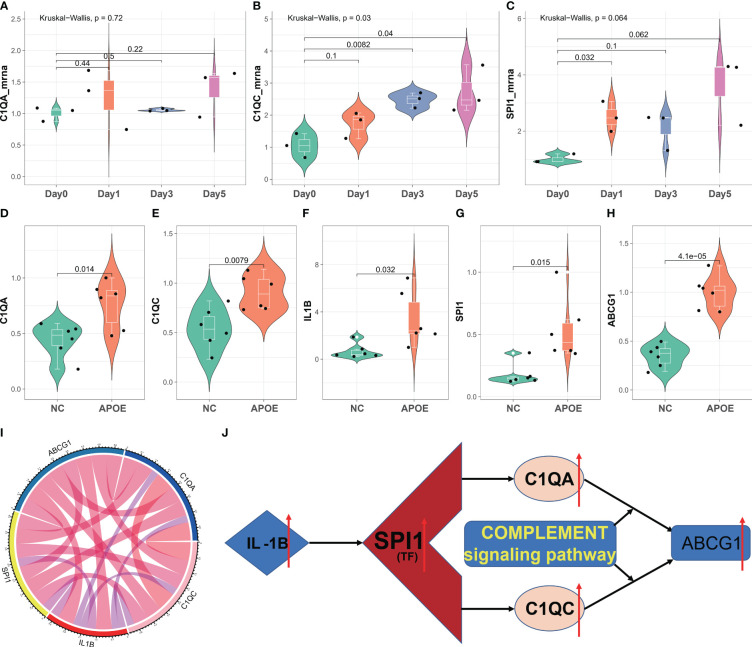
*In vitro* and *in vivo* validation of C1QA and C1QC. **(A–C)** Relative mRNA expression of C1QA and C1QC detected by real-time PCR in ox-LDL-treated RAW264.7 macrophages group and normal control group. **(D–H)** Relative mRNA expression value of C1QA, C1QC, IL1B, SPI1, and ABCG1 detected by real-time PCR in thoracic and abdominal arteries of apoE^-/-^ mice and normal mice. **(I)** The five genes were positive correlated with each other. **(J)** The potential mechanism for HAP development regulated by the C1QA and C1QC genes.

To validate our diagnostic prediction model, we conducted *in vitro* and *in vivo* experiments to confirm the biological function of C1QA and C1QC. *In vitro*, we upregulated the mRNA expression of C1QA (**Figure 11A**), C1QC (**Figure 11B**) and SPI1 (**Figure 11C**) in the ox-LDL-treated RAW264.7 macrophages group compared to the control group (RAW264.7 macrophages group treated with normal saline).

The results of the *in vivo* study revealed that compared to healthy control mice, the expression of C1QA (**Figure 11D**) and C1QC (**Figure 11E**) genes were upregulated in the thoracic and abdominal arteries of apoE-/- mice, as confirmed by RT-qPCR. Additionally, the expression of IL1B (**Figure 11F**), SPI1 (**Figure 11G**), and ABCG1 (**Figure 11H**) genes also showed upregulation in the thoracic and abdominal arteries of the apoE-/- mice as compared to the healthy control mice. Moreover, these five genes displayed a positive correlation with each other (**Figure 11I**). These findings imply that C1QA and C1QC may promote the migration and proliferation of ABCG1 in macrophage cells. Based on the findings depicted in **Figure 11J**, it is postulated that SPI1 may augment the expression of IL1B, thereby inducing an upregulation of the C1QA and C1QC genes. This, in turn, results in the accumulation of ABCG1 genes within macrophages, leading to a reduction in foam cell formation and atheroma development. Ultimately, this mechanism confers protection against atherosclerosis, aligning with previously published research (51, 52).

### MR analysis of associations between C1Q and ischemic stroke

**Figure 12:**
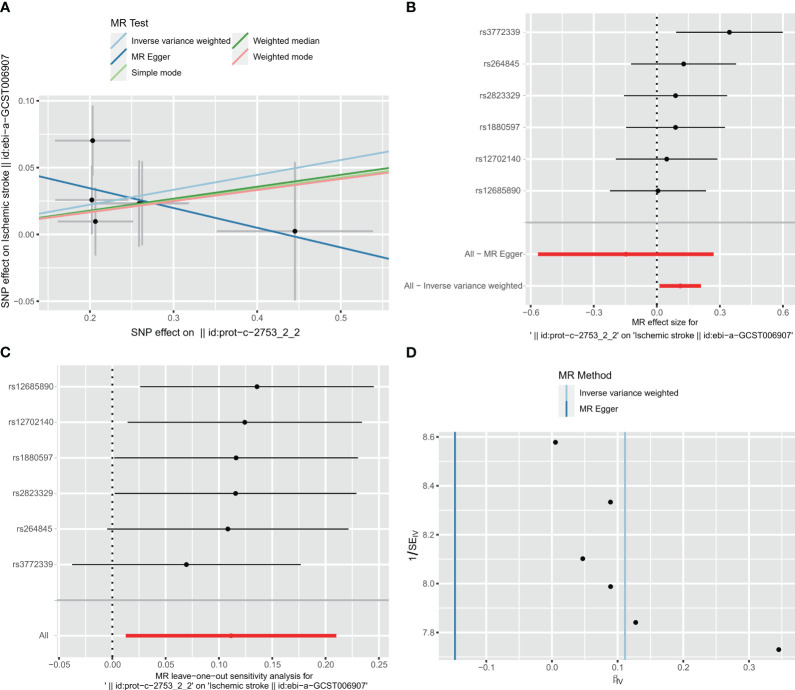
Visualization of the MR analysis of C1Q on ischemic stroke (IS). **(A)** Scatterplot of the MR analysis of the effect of C1Q on IS. **(B)** Forest plots of causal effects of C1Q-associated single nucleotide polymorphisms (SNPs) on IS. **(C)** Leave-one-out sensitivity analysis of the effect of C1Q on IS. **(D)** Funnel plot showed there were no significant heterogeneity among SNPs.

In the two-way Mendelian randomization (MR) analysis, six single nucleotide polymorphisms (SNPs) were selected to investigate the relationship between C1Q as the exposure and ischemic stroke (specifically, large artery atherosclerosis) as the outcome. The findings from the analysis revealed significant results for the inverse variance weighted (IVW) method (odds ratio [OR] = 1.118, 95% confidence interval [CI] = 1.013 - 1.234, p-value = 0.027), MR-Egger method (OR = 0.863, 95%CI = 0.568 - 1.31, p-value = 0.527), and weighted median method (OR = 1.093, 95%CI = 0.964 - 1.24, p-value = 0.1665). These results suggest that individuals with C1Q are genetically predisposed to a 1.118 times higher risk of ischemic stroke compared to those without C1Q, indicating a positive association between C1Q and ischemic stroke (**Table S9** and **Figure 12**). The Cochran’s Q report indicated the absence of heterogeneity among the independent variables (IVs) (**Table S10**, P>0.05), suggesting no heterogeneity among these IVs. The scatter plots in **Figure 12A** display the SNP effect sizes for C1Q and ischemic stroke (large artery atherosclerosis). The MR-Egger analysis revealed no evidence of horizontal pleiotropy (C1Q on risk of IS: intercept = 0.0641, P = 0.2795, **Table S11** and **Figure 12B**). The leave-one-out analysis identified high-impact points in 83.3% (5/6) of SNPs (P < 0.05), indicating that the association between C1Q and IS was influenced by the collective action of multiple SNPs (**Table S12** and **Figure 12C**). The results support a positive genetic relationship between C1Q and IS (large artery atherosclerosis).

### Reverse MR analysis of ischemic stroke on C1Q

**Figure 13:**
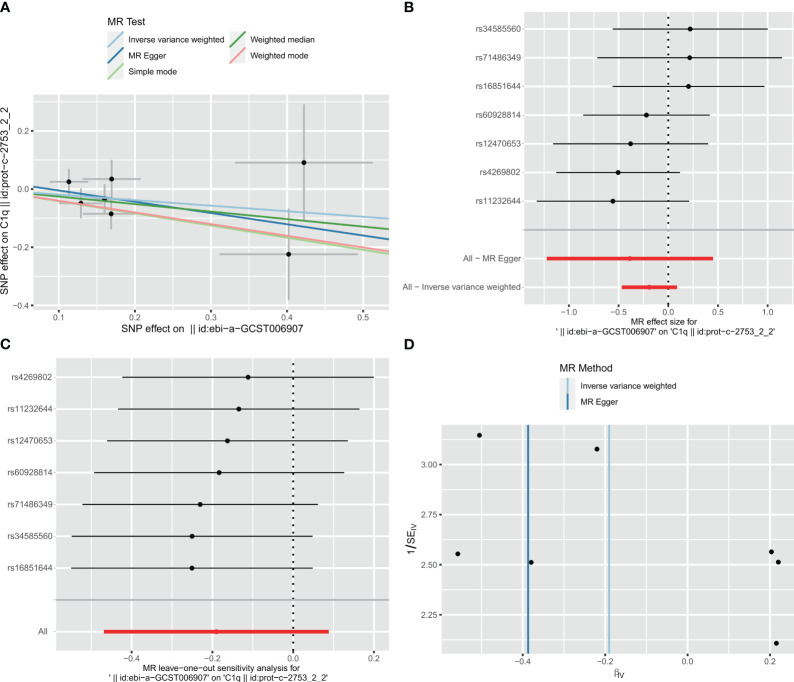
Visualization of the MR analysis of ischemic stroke (IS) on C1Q. **(A)** Scatterplot of the MR analysis of the effect of IS on C1Q. **(B)** Forest plots of causal effects of IS-associated single nucleotide polymorphisms (SNPs) on C1Q. **(C)** Leave-one-out sensitivity analysis of the effect of IS on C1Q. **(D)** Funnel plot showed there were no significant heterogeneity among SNPs.

Taking IS (large artery atherosclerosis) as exposure and C1Q as outcome, a total of 77 single nucleotide polymorphisms (SNPs) were extracted for analysis. The results of the analysis using the inverse variance weighted (IVW) method showed an odds ratio (OR) of 0.827 with a 95% confidence interval (CI) of 0.626 – 1.092, and a p-value of 0.181. The MR-egger method yielded an OR of 0.697 with a 95% CI of 0.294 – 1.569, and a p-value of 0.4067. The weighted median method resulted in an OR of 0.773 with a 95% CI of 0.529 – 1.128, and a p-value of 0.1817. These findings indicate that there was no significant correlation between ischemic stroke and C1Q, as shown in **Table S9** and **Figure 13A**. The Cochran’s Q report indicated the absence of heterogeneity among the independent variables (IVs) (**Table S10**, P>0.05), suggesting no heterogeneity among these IVs. The MR-Egger analysis revealed no evidence of horizontal pleiotropy (IS on risk of C1Q: intercept = 0.0338, P = 0.6462) (**Table S11** and **Figure 13B**). The leave-one-out analysis results demonstrated no significant abnormalities (**Table S12** and **Figures 13C, D**). Therefore, the findings do not support a reverse genetic relationship between C1Q and IS (large artery atherosclerosis).

## Discussion

Atherosclerotic lesions comprise cells generated by innate and adaptive immunity, which exert a substantial influence on the modulation of diverse immune cells during the evolution and advancement of atherosclerotic lesions (47). The utilization of scRNA-seq has facilitated the exploration of the molecular characteristics of immune cells infiltrating high plaque areas, as well as the roles of C1Q-related genes during the course of atherosclerosis. In the initial phase of this investigation, we performed scRNA-seq analysis of HAP and contiguous tissues to discern the subpopulations of cells present in HAP, and demonstrated the presence of C1Q cell cluster in HAP tissue. We then extracted the top 10 C1Q-related genes from C1Q cell cluster. We identified seven significant DEGs between AC and PA from scRNA-seq analysis. The GBM, LASSO and XGBoost algorithms were employed to create a diagnostic prediction model assigning GLM regression, which identified C1QA and C1QC as suitable C1Q hub genes for predicting the diagnosis of HAP. We further investigated SPI1 as a potential key transcription factor that regulates C1QA and C1QC in HAP and found that C1QA and C1QC were interdependent and activated by HALLMARK_COMPLEMENT signaling pathway. Furthermore, qPCR analysis confirmed the upregulated expression of C1QA, C1QC, SPI1, IL1B, and ABCG1 genes in the thoracic and abdominal arteries of apoE-/- mice. Finally, bidirectional mendelian randomization analysis conducted on the IEU open GWAS data revealed a positive correlation between C1Q and HAP (ischemic stroke (large artery atherosclerosis)). Upon successful validation, it is expected that the two hub genes associated with C1Q could serve as valuable diagnostic tools and provide guidance for the development of immunotherapeutic strategies in patients with HAP.

In the HAP, the scRNA-seq has found immune cells in plaques to be associated with cerebrovascular events (17, 19, 20, 22), while bioinformatics analyses have confirmed that immune cell infiltrations and immune-associated pathways (IAP) play a role in AP development (7–14). In this study, 8 of 12 cell clusters, including CT4+ T, CT8+ T, macrophages, monocytes, dendritic cells, B cells, mast cells and plasma cells, were immune cells from scRNA-seq analysis of GSE159677 data. In AC groups, immune cells represented more than 70% of the total cells, whereas in adjacent groups, they accounted for less than 45%. The GSEA showed that the most common signaling pathways, such as the immune signaling (INTERFERON_GAMMA_RESPONSE, IL6_JAK_STAT3_SIGNALING and IL2_STAT5_ SIGNALING), allograft rejection, the complement response, the inflammatory response, metabolism and glycolysis were enriched in HAP tissues. These observations unveil the pivotal part played by the immune system in the pathogenesis of atherosclerosis.

When compared with the adjacent tissues in GSE159677, we found that the proportion of T cells increased from 29.39% to 40.08%, while the percentage of macrophages increased from 5.17% to 17.94%. There was a remarkably high level of upregulation of both C1QA and C1QC across all scRNA-seq and bulk-RNA datasets observed as atherosclerosis progressed. The ROC curves showed that both C1QA and C1QC were able to distinguish HAP samples from adjacent/normal tissues. The findings of our study indicate that the immune marker genes we have identified may have significant implications in the pathogenesis of atherosclerosis. Conducting further research on the disease-associated molecular processes and immune cell regulation may facilitate the development of potential therapeutic interventions.

**Figures 3A–C** shows the scRNA-seq analysis results indicating that C1QA and C1QC were predominantly expressed in macrophages. Consistent with previous findings, Castellano G et al. have confirmed that dendritic cells derived from monocytes and macrophages are the main producers of C1Q (53). Therefore, our study’s two marker genes align with these prior observations. Given that macrophages constitute a major immune cell population in atherosclerosis (54, 55), with macrophages playing a significant role, we selected them for functional experiments at the cellular level.

C1Q, a classical component of the complement system, can perform immunological and non-immune functions, either complement-dependent or complement-independent (56). The complement system’s effects can be either beneficial or harmful, contingent upon the pathophysiological mechanisms at play, and in certain instances, it may cause tissue damage (57). Research has demonstrated that C1Q plays a protective role in the initial phases of neuronal injury and amyloid-induced neurotoxicity by suppressing inflammation (58). Additionally, during cholesterol ingestion, C1Q can promote macrophage survival and enhance foam cell efferocytosis function, suggesting a possible protective role in the early stages of atherosclerosis (59, 60).

Previous research has indicated a positive correlation between C1Q and coronary artery disease, with the potential for it to serve as a cardiovascular event indicator (61, 62). Our own investigation supports this finding, as heightened C1Q expression may significantly contribute to atherosclerotic plaque instability or rupture. Additionally, Chen LH et al. have proposed that groups exhibiting elevated levels of C1QA, C1QB, and C1QC display notably enriched signaling pathways associated with immune functions, such as allograft rejection, complement response, and inflammatory response (56). Our research revealed that C1QA and C1QC were significantly overexpressed in HAP and were also enriched in signaling pathways of allograft rejection, the complement response, and the inflammatory response. Additionally, we confirmed that macrophage-derived foam cells had overexpression in RAW264.7 macrophages treated with oxLDL. Therefore, the confirmed overexpression of C1QA and C1QC in atherosclerotic plaques indicated that these two markers are correlated with plaque macrophages.

Previous research has examined the correlation between HAP immune scores and diagnostic predictions for patients with TNFSF13B, CCL5, CCL19, ITGAL, CD14, GZMB, and BTK genes, which were utilized as predictive targets (9). For clinicians, the immune score is a reliable tool for predicting the progression of atherosclerotic plaques. In another comparable study, it was found that C1QA and ITGB2 could have pathogenic effects on the complete atherogenic process (13). In a recent study, Li et al (14) proposed five innovative diagnostic biomarkers for atherosclerosis based on oxidative stress and macrophage ferroptosis and confirmed them using GSE100927 and atherosclerosis tissues from animals. The primary theme in all three studies was the selection of hub genes utilizing the protein-protein interaction (PPI) network. The hub genes obtained may vary based on the datasets. In this study, we established hub genes on DEGs using scRNA-seq and two GEO datasets, which reduced the inconsistency in hub genes and improved the reliability of the outcomes. We obtained an AUC value similar to the three scholars with two immune genes (9, 13, 14).

It is remarkable that SPI1 might be the key transcription factor regulating the C1QA and C1QC genes in HAP pathology. As HAP progresses, IL1B and/or S100A8 are released (15, 63), inducing the growth of SPI1 and further upregulating C1QA and C1QC expression. As a consequence, excessive amounts of free cholesterol accumulate in macrophages as lipid droplets, leading to the formation of foam cells (high expression of ABCG1, etc.) (64–66). Our study showed that C1QA, C1QC, SPI1, IL1B, and ABCG1 expressions were all upregulated, not only in GEO datasets but also in apoE-/- mice thoracic and abdominal arteries. The elevated SPI1, stimulated by inflammatory cytokines, increases C1QA and C1QC, which ultimately leads to the formation of foam cells in macrophages. The hypothesis needs to be tested further with basic cellular and animal experiments. Additionally, we observed that the expression of C1QA and C1QC genes was positively correlated with the HALLMARK_COMPLEMENT signaling pathway in the analysis of three GEO datasets. Therefore, SPI1 may be upregulating C1QA and C1QC through the HALLMARK_COMPLEMENT pathway.

Our study has demonstrated an association between C1Q and an increased risk of HAP; however, Mendelian randomization studies do not provide supporting evidence for this relationship. To address this issue, biodirectional Mendelian randomization (MR) utilizing data from genome-wide association studies (GWAS) was employed to evaluate causality in a potential exposure-outcome pathway. In this particular investigation, C1Q was considered as the exposure variable, while ischemic stroke (specifically large artery atherosclerosis) was utilized as the outcome measure in place of HAP. To our surprise, the initial findings of the study revealed a statistically significant positive association between C1Q and IS, as indicated by the forward results (IVW: OR = 1.118, 95%CI = 1.013 -1.234, P = 0.027). This suggests that there is a genetic risk associated with C1Q and IS. Additionally, the leave-one-out analysis demonstrated that certain SNPs in C1Q have a substantial impact on IS (83.3% (5/6), P < 0.05). However, upon conducting a reverse MR analysis using the IVW and leave-one-out results, it was determined that there is no significant correlation between IS and C1Q. Despite this, the evidence gathered supports a positive genetic association between C1Q and IS, specifically in cases of large artery atherosclerosis.

Despite the identification of characteristic atherosclerotic plaque progression- and immune-associated genes through machine learning algorithms, which have been confirmed to be diagnostically effective in external datasets, this study is subject to certain limitations. In order to investigate the potential of these genes in predicting the progression of atherosclerotic plaques, prospective cohorts will need to be conducted. Additionally, to gain a more comprehensive understanding of the mechanisms underlying these characteristic genes, further experimentation is necessary. Ultimately, further studies are warranted to clarify the underlying mechanisms. Finally, the statistical measure of genetic aggregation constrains the analytical scope, while also accounting for interindividual variations.

In conclusion, our research has effectively established and validated a unique diagnostic signature comprising two C1Q-related marker genes by employing both single-cell and bulk RNA sequencing techniques. Furthermore, the utilization of MR analysis has confirmed a positive correlation between C1Q and HAP (ischemic stroke (large artery atherosclerosis)). The aforementioned signature exhibits considerable promise as a diagnostic biomarker and has the potential to enhance the prognostication of atherosclerosis progression. Additionally, our research has yielded valuable insights into the importance of C1Q-related hub genes in diagnosing and predicting the response to immunotherapy in patients with HAP.

## Data availability statement

The datasets presented in this study can be found in online repositories. The names of the repository/repositories and accession number(s) can be found in the article/**Supplementary Material**.

## Ethics statement

The animal studies were approved by Shanghai Sixth People’s Hospital Affiliated to Shanghai Jiao Tong University School of Medicine. The studies were conducted in accordance with the local legislation and institutional requirements. Written informed consent was obtained from the owners for the participation of their animals in this study.

## Author contributions

HC: Software, Writing – original draft, Writing – review & editing. CT: Writing – review & editing. YG: Data curation, Resources, Methodology, Writing – original draft. ZL: Data curation, Resources, Writing – review & editing. JZ: Writing – review & editing, Data curation, Investigation, Resources. YL: Formal Analysis, Software, Writing – review & editing.
