# Supplementary material for: An integrative analysis of single-cell and bulk transcriptome and bidirectional mendelian randomization analysis identified C1Q as a novel stimulated risk gene for Atherosclerosis
Source: Front Immunol. 2023 Dec 21;14:1289223. doi: 10.3389/fimmu.2023.1289223 (PMC10764496; doi:10.3389/fimmu.2023.1289223)
Supplement: Supplementary file 1 [file DataSheet_1.docx]

Supplementary Materials for

**An integrative analysis of single-cell and bulk transcriptome and** **bidirectional mendelian randomization analysis identified C1Q as a novel stimulated risk gene for** **Atherosclerosis**

Hong-Kai Cui ^1*^, MD; Chao-Jie Tang^2*^, MD; Yu Gao ^1^, MD; Zi-Ang Li ^1^, MD; Jian Zhang^1^, MD; Yong-Dong Li ^1,2^, MD, Ph.D.

**Institutional Affiliation**

1. Department of Neurological Intervention, The First Affiliated Hospital, Xinxiang Medical University, Xinxiang, Henan Province, China

2. Institute of Diagnostic and Interventional Radiology, Shanghai Sixth People's Hospital Affiliated to Shanghai Jiao Tong University School of Medicine

* Hong-Kai Cui and Chao-Jie Tang contributed equally to this work

**Corresponding Author**. Hong-Kai Cui & Yong-Dong Li

**Address**.  No.88, Jian Kang Road, Weihui, Henan Province

No. 600, Yi Shan Road, Shanghai, 200233, China

**E-Mail Address**. [chk-1980@163.com](mailto:chk-1980@163.com) & dr_liyongdong@sina.com

**Postal code**. 453100

**Phone**. 0086-0371-65165352

**Fax**. 0086-0371-66971086

**This file includes:**

Figs. S1 to S6

Tables S1-3，S7-12

**Supplementary Figures**


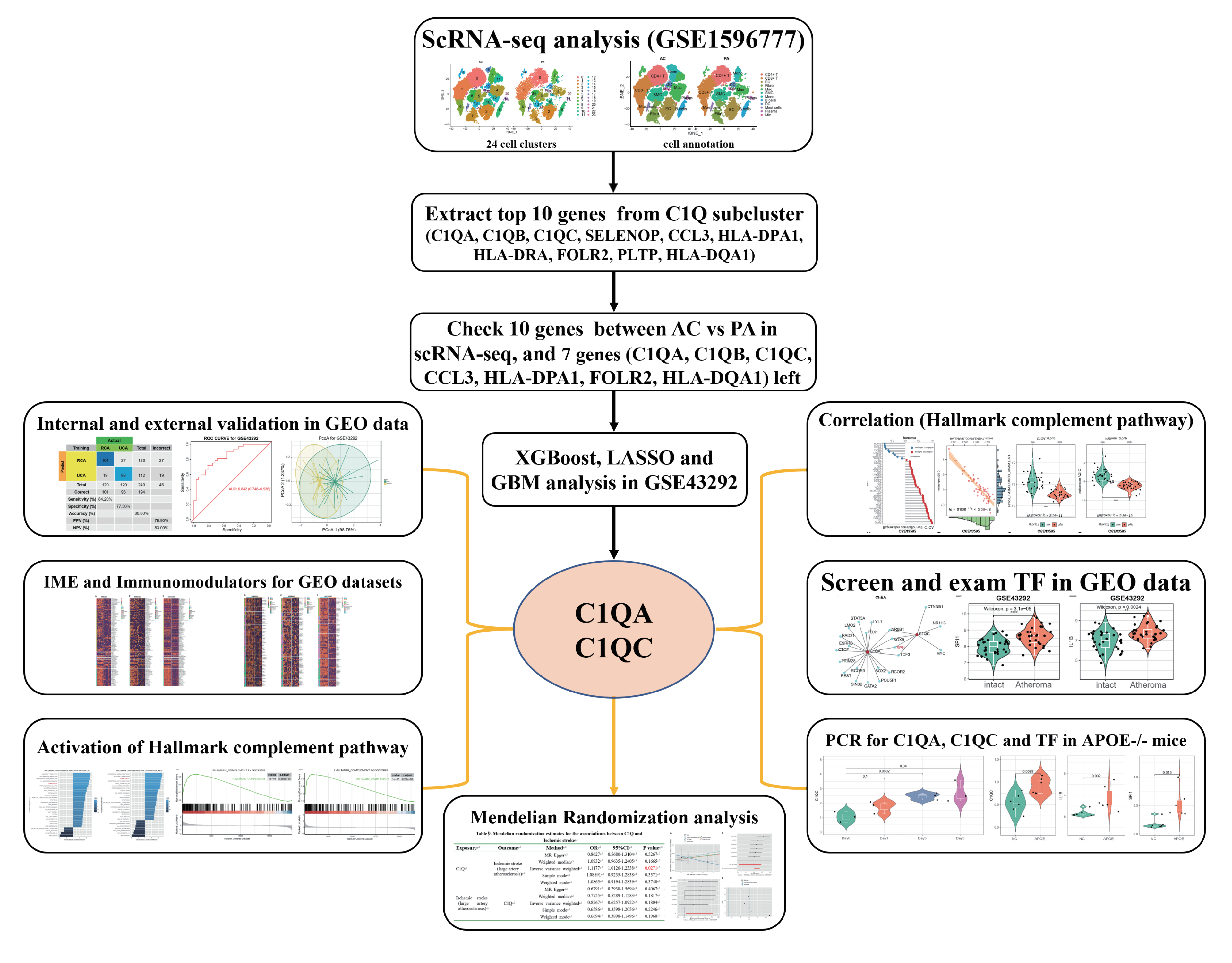


**Fig. S1. Workflow of the study.**

**Note: DEG** = Differentially expressed genes; **LASSO** = Least absolute shrinkage and selection operator; **TF** = Transcription factor; **PCR** = Polymerase chain reaction; **AC** = Atherosclerotic core; PA = Proximally adjacent.


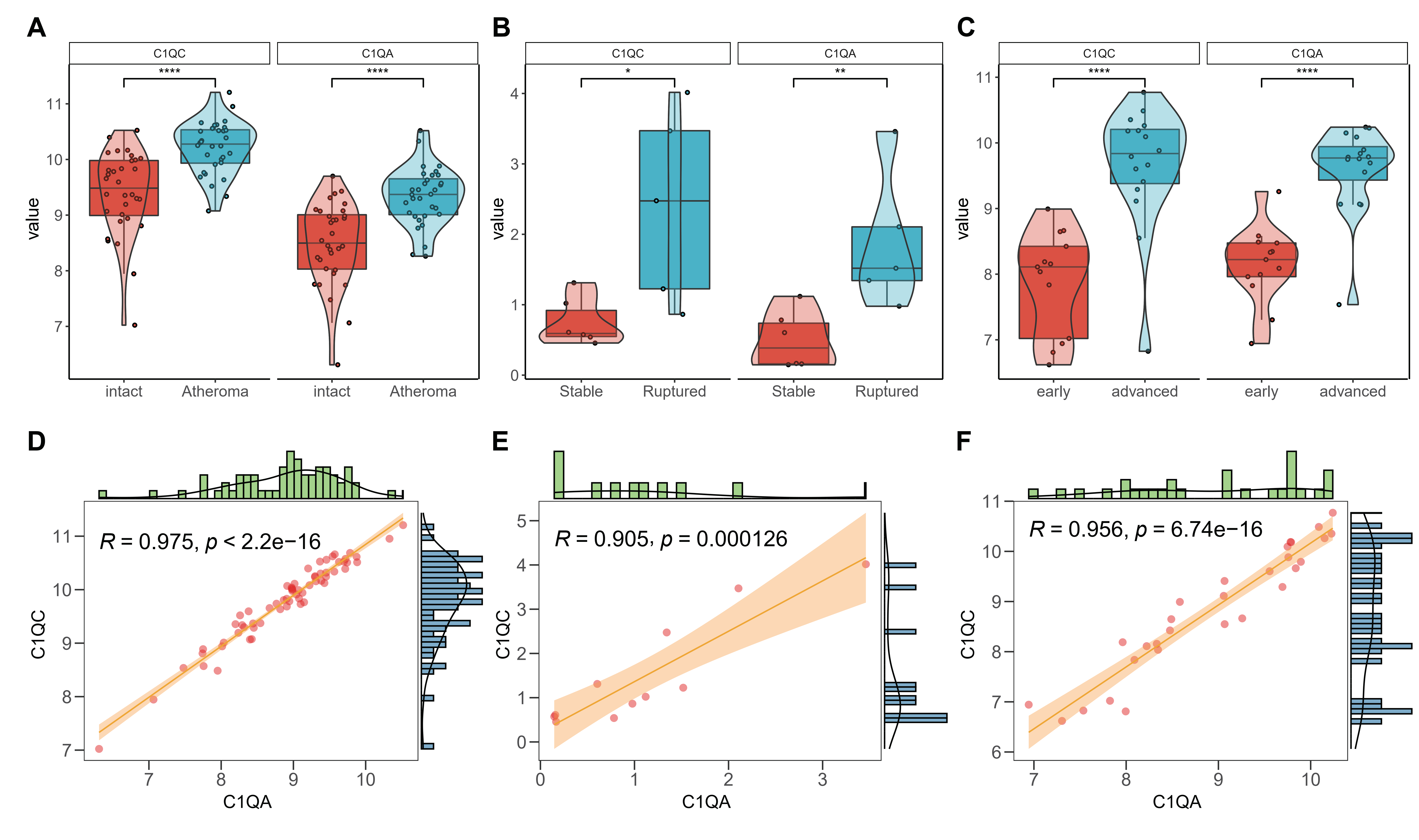


**Fig. S2.** The expression and correlations of the two biomarkers in the three GEO datasets. The expression of the two biomarkers in the GSE43292 **(A**), GSE41571 **(B**), GSE28829 **(C**) datasets. The correlations of the two biomarkers in the GSE43292 **(D**), GSE41571 **(E**), GSE28829 **(F**) datasets.


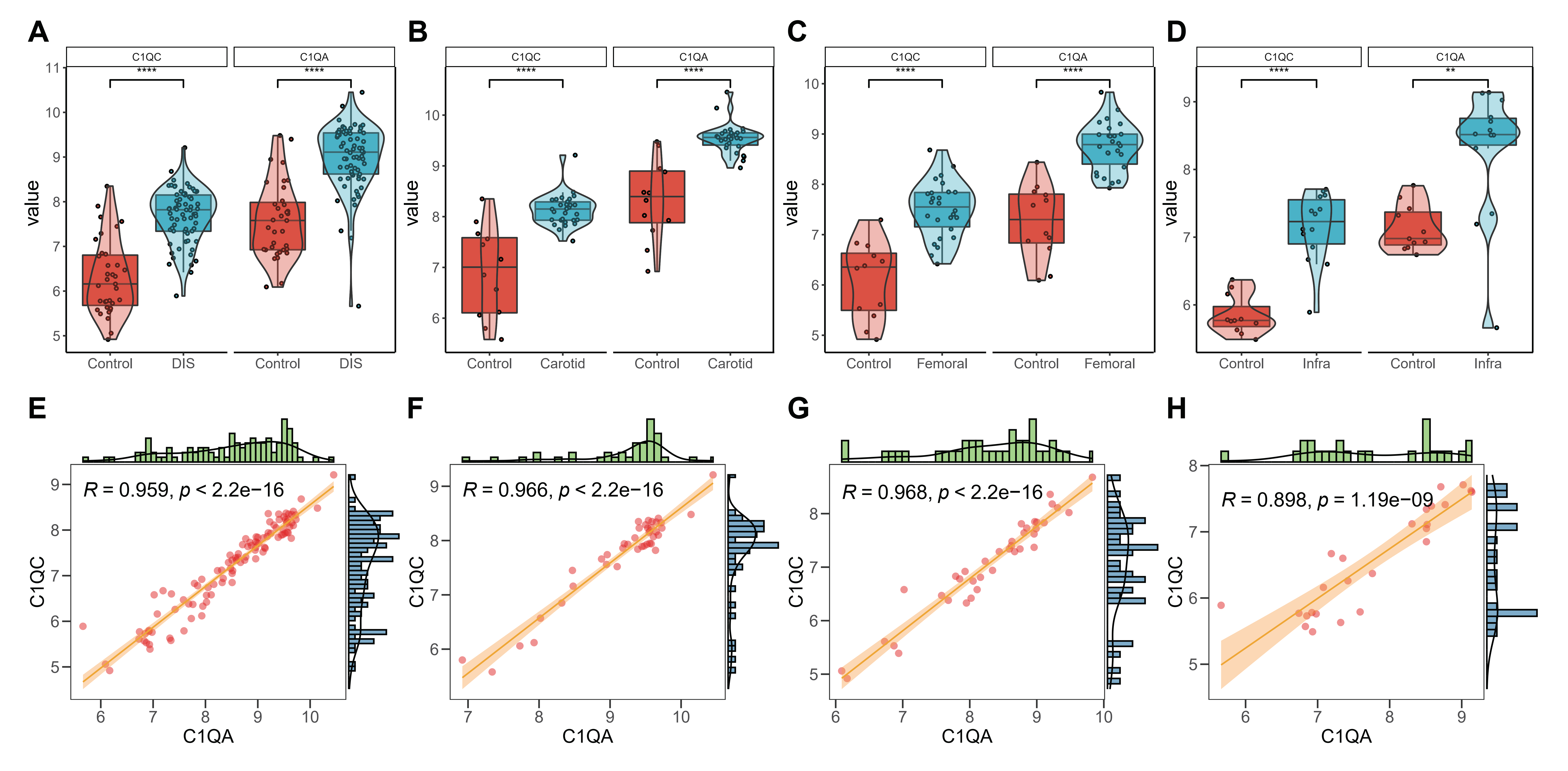


**Fig. S3**. The expression and correlations of the two biomarkers in the GSE100927 and its three subgroup datasets. The expression of the two biomarkers in the GSE100927 **(A**), GSE100927_Carotid **(B**), GSE100927_Femoral **(C**) and GSE100927_Infra **(D**) datasets. The correlations of the two biomarkers in the GSE100927 **(E**), GSE100927_Carotid **(F**), GSE100927_Femoral **(G**) and GSE100927_Infra **(H**) datasets.





**Fig. S4**. **Immune checkpoint expression analysis**

(**A**) Box plots depicting the levels of immune checkpoint in early-stage and advanced-stage atherosclerotic plaques in GSE43292. (**B**) Box plots depicting the levels of immune checkpoint in early-stage and advanced-stage atherosclerotic plaques in GSE28829. (**C**) Box plots depicting the levels of immune checkpoint in early-stage and advanced-stage atherosclerotic plaques in GSE41571. (**D**) Box plots depicting the levels of immune checkpoint in normal arteries and atherosclerotic plaques in GSE100927. *p < 0.05; **p < 0.01; and ***p < 0.001.


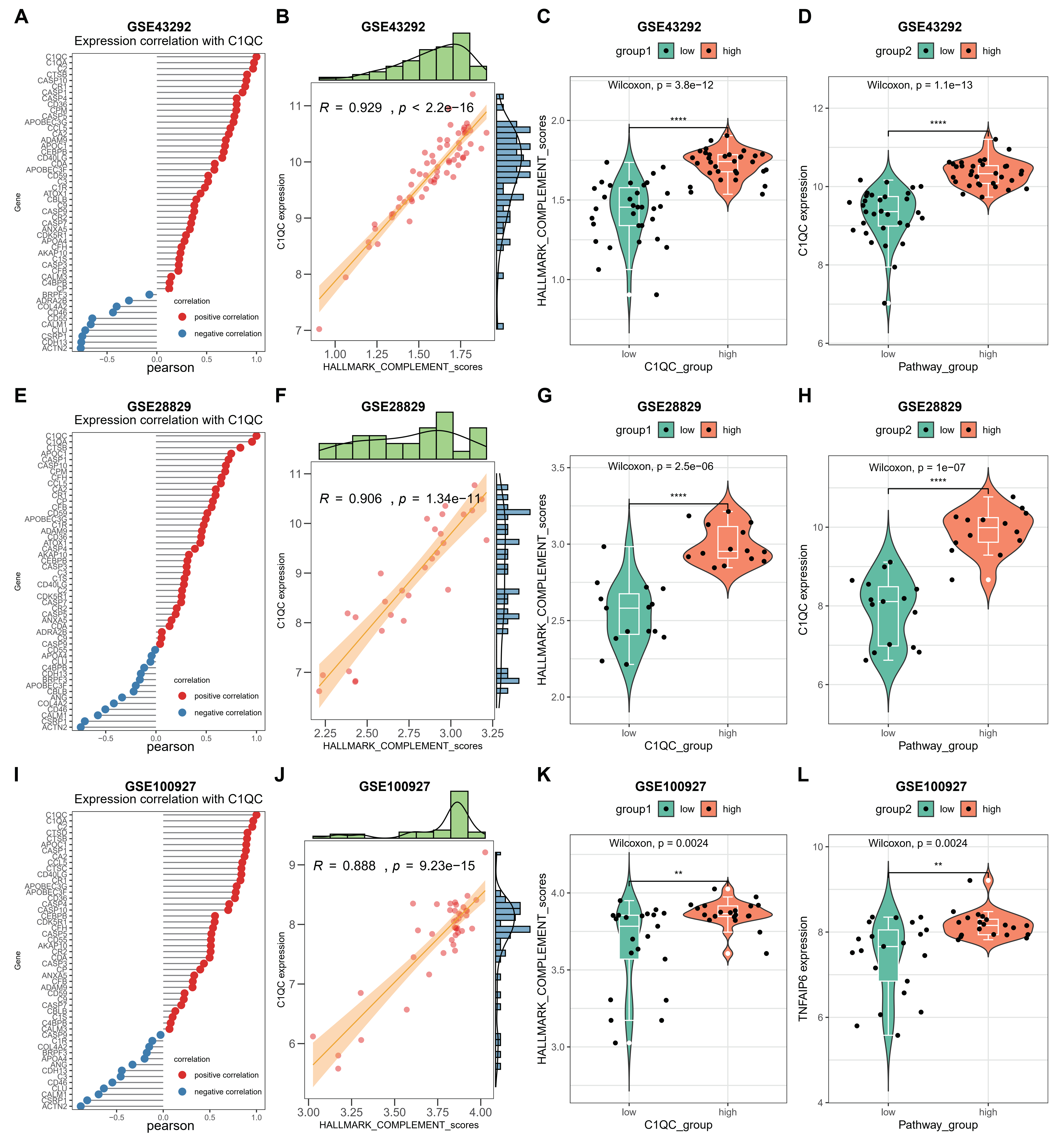


**Fig. S5**. **C1QC correlated HALLMARK_COMPLEMENT signaling pathway in HAP.**

(**A**) Correlation between C1QC and HALLMARK_COMPLEMENT signaling pathway genes in GSE43292 dataset. (**B**) Correlation between C1QC and HALLMARK_COMPLEMENT signaling pathway scores in GSE43292 dataset. (**C**) Comparison of the HALLMARK_COMPLEMENT signaling pathway scores between high and low C1QC expression subgroups in GSE43292 dataset. (**D**) Comparison of C1QC expression between high and low subgroups of HALLMARK_COMPLEMENT signaling pathway scores in GSE43292 dataset. (**E**) Correlation between C1QC and HALLMARK_COMPLEMENT signaling pathway genes in GSE28829 dataset. (**F**) Correlation between C1QC and HALLMARK_COMPLEMENT signaling pathway scores in GSE28829 dataset. (**G**) Comparison of the HALLMARK_COMPLEMENT signaling pathway scores between high and low C1QC expression subgroups in GSE28829 dataset. (**H**) Comparison of C1QC expression between high and low subgroups of HALLMARK_COMPLEMENT signaling pathway scores in GSE28829 dataset. (**I**) Correlation between C1QC and HALLMARK_COMPLEMENT signaling pathway genes in GSE100927 dataset. (**J**) Correlation between C1QC and HALLMARK_COMPLEMENT signaling pathway scores in GSE100927 dataset. (**K**) Comparison of the HALLMARK_COMPLEMENT signaling pathway scores between high and low C1QC expression subgroups in GSE100927 dataset. (**L**) Comparison of C1QC expression between high and low subgroups of HALLMARK_COMPLEMENT signaling pathway scores in GSE100927 dataset.


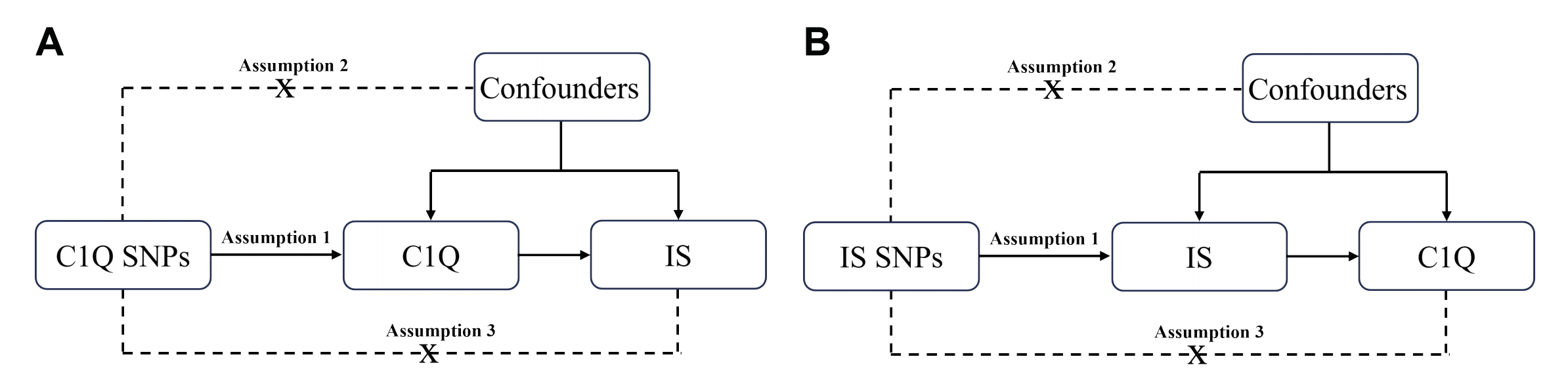


**Fig. S6**. **The schematic of the Mendelian randomization study of C1Q and IS.**

(**A**) C1Q SNPs were used as the genetic instruments to investigate the causal effect of C1Q on IS. (**B**) IS SNPs were used as the genetic instruments to investigate the causal effect of IS upon C1Q. Line with arrows indicates that the genetic instruments (SNPs) are associated with the exposure and could only affect the outcome via the exposure. Dashed lines indicate that the genetic instruments (SNPs) are independent of any confounding variables between the results. IS, ischemic stroke (large artery atherosclerosis)

**Supplementary Tables.**

**Table S1.** **The summary of the GEO datasets.**

| GEO ID | Platform | Tissue Type | Sample size | Experiment type |
| --- | --- | --- | --- | --- |
| GSE43292 | GPL6244 | Advanced carotid atherosclerotic plaques and early carotid atherosclerotic plaques | 32 vs. 32 | Array |
| GSE28829 | GPL570 | Advanced carotid atherosclerotic plaques and early carotid atherosclerotic plaques | 16 vs. 13 | Array |
| GSE41571 | GPL570 | Ruptures plaque andstable plaque | 5 vs. 6 | Array |
| GSE100927 | GPL17077 | Atherosclerotic arteries plaque and  healthy arteries | 69 vs. 35 | Array |

**Table S2.**  **50,856 cells from three patients were analyzed in this study.**

| ID | Cell counts | Percentage (%) |
| --- | --- | --- |
| Patient 1 AC | 10792 | 21.22 |
| Patient 1 PA | 3519 | 6.92 |
| Patient 2 AC | 15642 | 30.76 |
| Patient 2 PA | 5436 | 10.69 |
| Patient 3 AC | 12177 | 23.94 |
| Patient 3 PA | 3290 | 6.47 |

Note: AC = Atherosclerotic core; PA = Proximally adjacent

**Table S3. 44,843 cells from three patients were analyzed after** **initial quality control in this study.**

| ID | Cell counts | Percentage (%) |
| --- | --- | --- |
| Patient 1 AC | 9779 | 21.81 |
| Patient 1 PA | 3044 | 6.79 |
| Patient 2 AC | 13905 | 31.01 |
| Patient 2 PA | 4710 | 10.5 |
| Patient 3 AC | 10694 | 23.85 |
| Patient 3 PA | 2711 | 6.05 |

Note: AC = Atherosclerotic core; PA = Proximally adjacent

**Table S7. Characteristics of the 2 biomarkers and their coefficients in diagnostic prediction for AC vs. PA in GEO43292.**

| **Name** | **Coefficients** | **OR** | **z value** | **P value** |
| --- | --- | --- | --- | --- |
| Intersect | -24.304 | 2.7856e-11 | -3.468 | **< 0.001** |
| C1QA | 1.169 | 3.219e+0 | 1.950 | 0.5489 |
| C1QC | 1.339 | 4.0528e+0 | 2.051 | 0.4950 |

Note: AC = Atherosclerotic core; PA = Proximally adjacent

**Table S8.**  **Details of studies included in the Mendelian randomization analyses for the associations between C1Q and Ischemic stroke (large artery atherosclerosis)**

| **Parameter** | **Trait** | **Database** | **Year** | **Consortium** | **Population** | **Sex** | **SNP** | **Sample size** |
| --- | --- | --- | --- | --- | --- | --- | --- | --- |
| Exposure | C1Q | prot-c-2753_2_2 | 2019 | NA | European | Male and Female | 501428 | - |
| Outcome | Ischemic stroke (large artery atherosclerosis) | ebi-a-GCST006907 | 2018 | NA | European | NA | 8418349 | 410484 |

**Table S9. Mendelian randomization estimates for the associations between C1Q and Ischemic stroke**

| **Exposure** | **Outcome** | **Method** | **OR** | **95%CI** | **P value** |
| --- | --- | --- | --- | --- | --- |
| C1Q | Ischemic stroke (large artery atherosclerosis) | MR Egger | 0.8627 | 0.5680-1.3104 | 0.5267 |
|  |  | Weighted median | 1.0932 | 0.9635-1.2405 | 0.1665 |
|  |  | Inverse variance weighted | 1.1177 | 1.0126-1.2338 | 0.0271 |
|  |  | Simple mode | 1.08891 | 0.9235-1.2838 | 0.3571 |
|  |  | Weighted mode | 1.0865 | 0.9194-1.2839 | 0.3748 |
| Ischemic stroke (large artery atherosclerosis) | C1Q | MR Egger | 0.6791 | 0.2938-1.5694 | 0.4067 |
|  |  | Weighted median | 0.7725 | 0.5289-1.1283 | 0.1817 |
|  |  | Inverse variance weighted | 0.8267 | 0.6257-1.0922 | 0.1804 |
|  |  | Simple mode | 0.6586 | 0.3598-1.2056 | 0.2246 |
|  |  | Weighted mode | 0.6694 | 0.3898-1.1496 | 0.1960 |

**Table S10.**  **Heterogeneity of Wald ratios for the associations between C1Q and Ischemic stroke**

| **Exposure** | **Outcome** | **Q** | **df** | **P value** |
| --- | --- | --- | --- | --- |
| C1Q | Ischemic stroke (large artery atherosclerosis) | 2.888 | 4 | **0.5767** |
| Ischemic stroke (large artery atherosclerosis) | C1Q | 4.672 | 5 | 0.4572 |

**Table S11.**  **MR-Egger test for directional pleiotropy for the associations between C1Q and Ischemic stroke**

| **Exposure** | **Outcome** | **Intercept** | **SE** | **P value** |
| --- | --- | --- | --- | --- |
| C1Q | Ischemic stroke (large artery atherosclerosis) | 0.064 | 0.0512 | 0.2794 |
| Ischemic stroke (large artery atherosclerosis) | C1Q | 0.0338 | 0.0693 | 0.6462 |

**Table S12. Leave-one-out analysis for the associations between C1Q and Ischemic stroke**

| Exposure | Outcome | **SNP** | **B** | **SE** | **P-value** |
| --- | --- | --- | --- | --- | --- |
| C1Q | Ischemic stroke (large artery atherosclerosis) | rs12685890 | 0.135701438 | 0.05589079 | 0.015183391 |
|  |  | rs12702140 | 0.124231658 | 0.05605756 | 0.026681602 |
|  |  | rs1880597 | 0.116102023 | 0.058301893 | 0.04643762 |
|  |  | rs264845 | 0.108351272 | 0.057745705 | 0.060606953 |
|  |  | rs2823329 | 0.115618694 | 0.057830183 | 0.04557813 |
|  |  | rs3772339 | 0.069485763 | 0.054721323 | 0.204151799 |
|  |  | All | 0.111346808 | 0.050397975 | 0.027150249 |
| Ischemic stroke (large artery atherosclerosis) | C1Q | rs11232644 | -0.134608914 | 0.152491912 | 0.377383133 |
|  |  | rs12470653 | -0.162696189 | 0.152112292 | 0.284808626 |
|  |  | rs16851644 | -0.250599305 | 0.152584254 | 0.100514022 |
|  |  | rs34585560 | -0.250302718 | 0.152124089 | 0.099890409 |
|  |  | rs4269802 | -0.111699344 | 0.15885163 | 0.481951214 |
|  |  | rs60928814 | -0.183313747 | 0.157998175 | 0.2459564 |
|  |  | rs71486349 | -0.230305765 | 0.148932829 | 0.122014415 |
|  |  | All | -0.190312533 | 0.142096092 | 0.180465616 |
